# Deficiency in hereditary hemorrhagic telangiectasia-associated Endoglin elicits hypoxia-driven heart failure in zebrafish

**DOI:** 10.1242/dmm.049488

**Published:** 2023-06-02

**Authors:** Etienne Lelièvre, Charlotte Bureau, Yann Bordat, Maxence Frétaud, Christelle Langevin, Chris Jopling, Karima Kissa

**Affiliations:** ^1^ LPHI, INSERM, CNRS, Université de Montpellier, 34095 Montpellier, France; ^2^ Institut de Génomique Fonctionnelle, Université de Montpellier, CNRS, INSERM LabEx ICST, 34094 Montpellier, France; ^3^ INRAE, Université Paris-Saclay, VIM, 78350 Jouy-en-Josas, France; ^4^ INRAE, Université Paris-Saclay, IERP, 78350 Jouy-en-Josas, France

**Keywords:** Endoglin, HHT, Heart failure, Cardiomegaly, Hypoxia, Endothelial cells

## Abstract

Hereditary hemorrhagic telangiectasia (HHT) is a rare genetic disease caused by mutations affecting components of bone morphogenetic protein (BMP)/transforming growth factor-β (TGF-β) signaling in endothelial cells. This disorder is characterized by arteriovenous malformations that are prone to rupture, and the ensuing hemorrhages are responsible for iron-deficiency anemia. Along with activin receptor-like kinase (*ALK1*), mutations in endoglin are associated with the vast majority of HHT cases. In this study, we characterized the zebrafish *endoglin* locus and demonstrated that it produces two phylogenetically conserved protein isoforms. Functional analysis of a CRISPR/Cas9 zebrafish *endoglin* mutant revealed that Endoglin deficiency is lethal during the course from juvenile stage to adulthood. Endoglin-deficient zebrafish develop cardiomegaly, resulting in heart failure and hypochromic anemia, which both stem from chronic hypoxia. *endoglin* mutant zebrafish display structural alterations of the developing gills and underlying vascular network that coincide with hypoxia. Finally, phenylhydrazine treatment demonstrated that lowering hematocrit/blood viscosity alleviates heart failure and enhances the survival of Endoglin-deficient fish. Overall, our data link Endoglin deficiency to heart failure and establish zebrafish as a valuable HHT model.

## Teaser

**Summary:** CRISPR/Cas9-engineered Endoglin deficiency in zebrafish recapitulates critical aspects of hereditary hemorrhagic telangiectasia (HHT) and thus provides a valuable model for use in large-scale screens of HHT-active compounds.

## INTRODUCTION

Endoglin (CD105) belongs to the bone morphogenetic protein (BMP)/transforming growth factor-β (TGF-β) receptor superfamily, and binds TGF-β1, TGF-β3, BMP9 and BMP10 (Castonguay et al., 2011; Cheifetz et al., 1992). This single-pass transmembrane protein acts as an ancillary receptor by modulating the signaling activity of the heterotetrameric receptor complex constituted of type I and type II serine-threonine kinase receptors. These, in turn, can phosphorylate R-Smads, which subsequently associate with Co-Smad (Smad4) to form a complex that regulates the transcription of target genes via Smad-binding elements (Massagué, 2012). Endoglin is mainly expressed in endothelial cells (ECs), and endoglin-deficient mice die around embryonic day (E)10.5 from cardiovascular defects associated with improper vascular smooth cell coverage (Li et al., 1999). In addition to in ECs, in mammals, endoglin is expressed in neural crest cells and their derivatives, such as vascular smooth muscle cells responsible for blood vessel stabilization and vascular tone control (Mancini et al., 2007). Endoglin is also expressed in hematopoietic stem cells (HSCs) as they emerge from intra-aortic clusters to be later restricted to HSCs with long-term repopulating capacities (Chen et al., 2002).

In humans, inactivating mutations in the endoglin (*ENG*) gene are responsible for hereditary hemorrhagic telangiectasia (HHT)1, whereas HHT2 is due to mutations affecting *ALK1* (also known as *ACVRL1*), a type I TGF-β receptor specifically expressed in ECs. Together, HHT1 and HHT2 account for ∼85% of diagnosed HHT cases (McDonald et al., 2015). *ENG*- and *ALK1*-independent HHT cases are associated with mutations affecting *GDF2* (also known as *BMP9*, encoding a high-affinity ligand for ALK1), microprocessor RNAse III *DROSHA* and additional uncharacterized loci. Lastly, mutations in *SMAD4* are causative of juvenile polyposis-HHT (Gallione et al., 2006; Jiang et al., 2018; Wooderchak-Donahue et al., 2013).

HHT (also known as Rendu-Osler-Weber syndrome) is a rare inherited autosomal-dominant genetic disorder characterized by a wide array of symptoms, among which mucocutaneous telangiectasias and recurrent epistaxis are the prominent diagnostic cues. These angiodysplastic lesions or arteriovenous malformations (AVMs) also often affect organs such as the gastrointestinal tract, the lungs, the liver and the brain. These AVMs resulting from the loss of the arteriolar-capillary plexus connect venous vessels directly to arteries exposing them to arterial-type blood flow mechanics (Guttmacher et al., 1995). Although much concern arises from brain, liver or lung AVMs, which can have life-threatening outcomes, gastrointestinal hemorrhages or recurrent epistaxis are also important issues, resulting in iron-deficiency anemia that requires medical management (Stross, 2013).

Congestive heart failure, although uncommon, constitutes another critical clinical manifestation of HHT. Cases are mainly associated with liver AVMs and chronic anemia of iron-deficiency type (Cho et al., 2012; Goussous et al., 2009; Montejo Baranda et al., 1984; Wu et al., 2017).

Albeit different in terms of incidence in specific organs, all forms of HHT rely on mutations affecting genes associated with the BMP/TGF-β pathway, and recent findings regarding *DROSHA* indicate that non-classical BMP/TGF-β pathways are also involved (Letteboer et al., 2006). Although this pathway is indisputably at the core of the pathology, tissue-specific cues or vascular bed specificities provide a terrain that somehow triggers or promotes the onset of the pathology. In humans, HHT relies on hemizygous mutations, and, accordingly, mice carrying a single knockout *Eng* allele spontaneously develop HHT-like phenotypes with varying penetrance depending on the genetic background (Bourdeau et al., 1999). This is collectively referred to as the ‘second hit’ concept by which additional alterations would be required for the development of the pathology. Consistent with this concept, angiogenic/inflammatory environments are potent drivers of AVM formation in heterozygous *Eng* and *ALK1* knockout mice (Tual-Chalot et al., 2015).

Several recent studies using BMP9/10 blocking antibodies, inducible tissue-specific *Eng*, *ALK1* and *Smad4* knockout mouse models as well as *endoglin* genome editing in zebrafish have provided important insights into the cellular and molecular mechanisms governing AVM formation. This involves inappropriate signaling responses to blood flow affecting EC polarization, venous/arterial identity maintenance, proliferation and pericyte recruitment (Baeyens et al., 2016; Jin et al., 2017; Ola et al., 2016, 2018; Sugden et al., 2017).

In this study, we examined the consequences of targeted *endoglin* disruption in postembryonic-stage zebrafish. Using a CRISPR/Cas9 *endoglin* mutant, we demonstrate that homozygous mutants die from congestive cardiomyopathy, accompanied by iron-deficiency anemia, from 1 month postfertilization. We found that this pathological condition sets in just before 15 days postfertilization (dpf), with an increase in hypoxia and cardiac stress markers in conjunction with heart chamber enlargement (cardiomegaly). In addition to HHT-like vascular lesions in organs such as the brain, mutant zebrafish exhibit structural alterations of the gill, strongly suggesting that the associated hypoxia stems from improper gill functioning. Chronic hypoxia stimulates erythropoiesis, which increases hematocrit. Hematocrit/blood viscosity management using the hemolytic agent phenylhydrazine (phz) markedly reduces cardiac stress and results in enhanced survival. Thus, by reproducing important features of HHT, our zebrafish model of endoglin deficiency lays the ground for future detailed molecular analysis that will be important for both the identification of HHT altered signaling pathways and the development of new treatments.

## RESULTS

### Zebrafish *endoglin* locus, transcripts and expression characterization

**Fig. 1.**
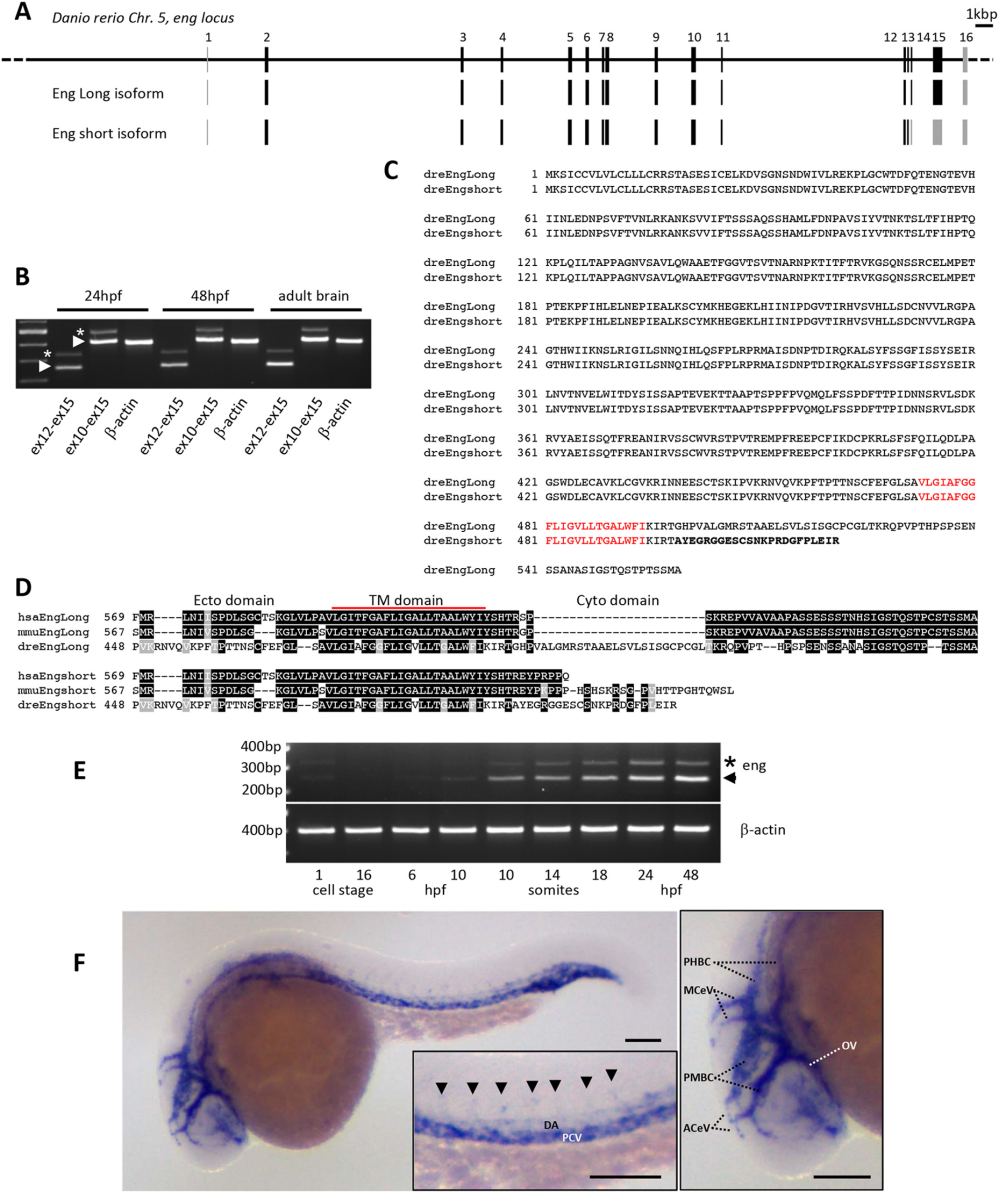
**Characterization of zebrafish *endoglin* chromosomal organization, transcripts and expression pattern.** (A) Zebrafish *endoglin* gene exon-intron structure on chromosome 5. Coding exons (black) and non-coding exons (gray) are shown. (B) RT-PCR analysis of *endoglin* variants. Endoglin long (arrowheads) and short (asterisks) isoform messenger expression in embryo and adult tissue (brain). (C) Protein alignment of zebrafish Endoglin isoforms. Alternative amino acids (aa) of Endoglin short isoform are in bold. Transmembrane aa are in red. (D) Alignment of human, mouse and zebrafish Endoglin short isoform C-terminal region. Identical (black) and similar (gray) aa are shown. TM, transmembrane. (E) RT-PCR analysis of *endoglin* variants during zebrafish development. Endoglin short isoform (asterisk) and Endoglin long isoform (arrowhead) are shown. hpf, h postfertilization. (F) Left: whole-mount *in situ* hybridization using *endoglin* antisense riboprobe on 24 hpf wild-type embryo. Inset: close-up of the trunk region showing differential *endoglin* expression between the dorsal aorta (DA) and the posterior cardinal vein (PCV). Arrowheads indicate intersegmental vessels. Right: close-up of the head region showing mostly venous *endoglin* expression. ACeV, anterior cerebral vein; MCeV, middle cerebral vein; PHBC, primordial hindbrain channel; PMBC, primordial midbrain channel; OV, optic vein. Scale bars: 100 µm.

In order to develop tools to address Endoglin expression and function in zebrafish, we first characterized the *endoglin* locus and associated transcripts in this organism. We conducted 5′ and 3′ rapid amplification of cDNA ends (RACE) experiments and obtained complete sequence data, which allowed us to reconstruct the exon-intron organization of the zebrafish *endoglin* gene. We identified three previously undescribed exons (Fig. 1A). Two are non-coding exons: one very-short exon is located at the utmost 5′ positioning *endoglin* transcription start site (TSS), and the other, located in 3′, contains most of the *endoglin* 3′ untranslated region (UTR). The third exon we identified (exon 13) corresponds to an alternative exon that introduces an early stop codon, resulting in an Endoglin protein isoform with a very short cytosolic domain (Fig. 1B,C). Interestingly, this isoform is also expressed in mammals (Bellón et al., 1993). In contrast to long isoforms, phylogenetic alignments revealed little or no conservation between species in the cytosolic tail of short isoforms (Fig. 1D). Reverse transcription PCR (RT-PCR) analysis indicated that both variants are expressed in embryonic and adult tissues; however, *endoglin* messenger for the short isoform is markedly less abundant than that for the long isoform (Fig. 1B). Similar analysis performed on developmentally staged embryos showed that, with exception of a marginal maternal contribution, the zygotic expression of *endoglin* coincides with early somitogenesis and gradually expands at later stages, with a relatively stable long isoform/short isoform ratio (Fig. 1E). Whole-mount *in situ* hybridization demonstrated that *endoglin* is expressed in developing blood vessels and mostly associated with veins (Fig. 1F). Thus, conserved synteny, EC-specific expression, sequence homology and similar protein isoforms together suggested that Endoglin function is conserved throughout the whole vertebrate phylum.

### Endoglin-deficient zebrafish die from congestive heart failure

**Fig. 2.**
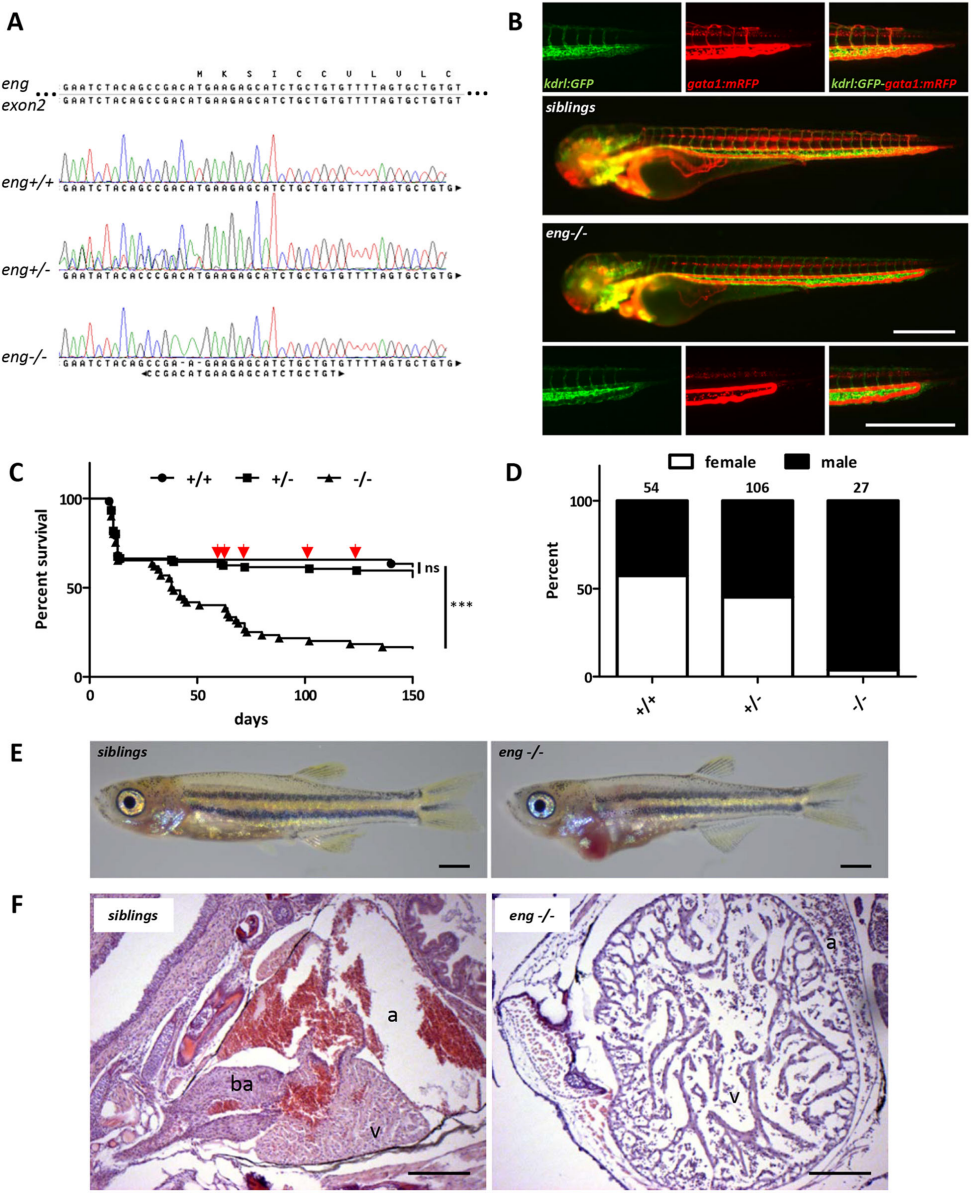
**Endoglin deficiency results in congestive heart failure in zebrafish.** (A) Sanger sequencing chromatograms of gRNA-targeted region on *endoglin* exon 2 in wild-type (*eng^+/+^*), heterozygous (*eng^+/−^*) and homozygous (*eng^−/−^*) mutant zebrafish. Complementary gRNA sequence is indicated below chromatograms. (B) Imaging of 72 hpf sibling and *eng^−/−^* zebrafish blood vessel perfusion. Analysis performed in *Tg(kdrl:GFP), Tg(gata1:mRFP)* background to highlight blood vessels and erythrocytes, respectively. Images are representative of data from siblings (*n*=10) and *eng^−/−^* fish (*n*=8). Scale bars: 500 µm. (C) Kaplan–Meier representation of *eng^+/+^*, *eng^+/−^* and *eng^−/−^* fish survival. +/+ versus −/− and +/− versus −/−, ***P<0.0001; +/+ versus +/−, not significant (ns) [log-rank (Mantel–Cox) test]. +/+, *n*=45; +/−, *n*=104; −/−, *n*=56. Arrows point to symptomatic siblings later identified as *eng^+/−^*. (D) Influence of genotype over sex ratio in individuals aged 3 months and above. Total number of individuals analyzed is indicated above bars. (E) Representative morphology of 30 dpf sibling and *eng^−/−^* fish. Note the enlarged cardiac area and overall paleness in *eng^−/−^* fish. Scale bars: 1 mm. (F) H&E-stained heart histological sections reveal enlargement and structural alteration of the ventricle, hypochromic red blood cells and swollen surrounding tissue. a, atrium; ba, bulbus arteriosus; v, ventricle. Scale bars: 100 µm.

To address Endoglin function in zebrafish, we used a CRISPR/Cas9 approach based on a guide RNA (gRNA) overlapping with *endoglin* ATG located on exon 2. In this manner, we generated a fish line carrying a unique indel consisting of a 2 bp deletion and a C>A or T>A base change, which destroyed the *endoglin* start codon and nucleotide −1 and −2 of the Kozak sequence (Fig. 2A). Analysis of *endoglin* knockout (*eng^−/−^*) zebrafish indicated that they developed and established blood circulation normally and were indiscernible from wild-type (*eng^+/+^*) and heterozygous (*eng^+/−^*) siblings. By 3 dpf, however, *eng^−/−^* exhibited a strong modification of blood flow pattern in a simple dilated loop formed by the dorsal aorta and the posterior cardinal vein. In this loop, blood flow was considerably accelerated and abnormally pulsatile in the venous part, while intersegmental vessels were poorly perfused (Fig. 2B), as described previously (Sugden et al., 2017). In clutches from heterozygous incrosses, fish with mutant phenotype were observed at a 25% frequency, indicating that this phenotype was fully penetrant (see Fig. 6B). Whole-mount *in situ* hybridization experiments showed a mild decrease in *endoglin* expression in mutants, and quantitative RT-PCR (RT-qPCR) revealed that *endoglin* mRNA abundance was reduced by 36.6% and 26.2% in homozygous mutants (*eng^−/−^*) compared to wild type and siblings, respectively (Fig. S1A,B), which contrasts with the strong nonsense-mediated mRNA decay (NMD) observed in a previous study (Sugden et al., 2017). *endoglin* mutation effect was evaluated in survival experiments from 3 dpf onwards. Kaplan–Meier survival plots revealed a high mortality rate associated with *eng^−/−^* fish (Fig. 2C). Indeed, although 63.5% of *eng^+/+^* and 59.6% of *eng^+/−^* zebrafish survived up to 5 months, only 16.8% of *eng^−/−^* zebrafish reached this age. Plots indicated that *eng^−/−^* zebrafish started to die around 1 month of age and displayed a median survival of 44 days. A fraction of *eng^−/−^* fish survived for the 5-month test period, but survival figures underestimated the severity of the phenotype as close to half (4/10) of the *eng^−/−^* fish never reached adulthood, which precluded sex determination. Surprisingly, *eng^−/−^* individuals reaching adulthood were found to be almost exclusively males (Fig. 2D). Because zebrafish sex is not assigned chromosomally, this could reflect either a genuine bias towards males or a higher sensitivity of females to Endoglin deficiency. Examination of *eng^−/−^* zebrafish at 30 dpf (the onset of lethality) revealed that most fish exhibited a red enlarged cardiac area, whereas the rest of the body appeared paler than that of siblings (Fig. 2E). Also, in contrast to siblings, *eng^−/−^* zebrafish were hyperventilating and displayed a marked surface respiratory behavior (SRB). To gain a better insight into this cardiac phenotype, we analyzed histological sections of the cardiac region (Fig. 2F). From these, the ventricle of *eng^−/−^* zebrafish appeared dramatically oversized. Red blood cells in *eng^−/−^* zebrafish did not present the characteristic orange color resulting from Eosin reaction with hemoglobin, reflecting a decrease in hemoglobin content. The surrounding connective tissue also appeared loose, indicating that edema, an accompanying symptom of heart dysfunction, was also taking place. Altogether, these data demonstrated that Endoglin-deficient zebrafish develop lethal congestive heart failure and anemia.

### A single *endoglin* mutant allele is sufficient to induce pathology

Based on macroscopic analysis, from 3 months onwards, surviving *eng^−/−^* zebrafish fell into three classes of phenotype severity: (1) arrested growth, hyperventilation and enlarged cardiac area; (2) normal size, hyperventilation and enlarged cardiac area to various degrees; and (3) no observable phenotype (asymptomatic) (Fig. S2). Interestingly, a fraction of *eng^+/−^* zebrafish also displayed a hyperventilation phenotype occasionally associated with an enlarged cardiac area (Fig. S2). Collective data from two independent experiments indicated that the frequency of *eng^+/−^* zebrafish presenting a phenotype averaged 14% of total *eng^+/−^* zebrafish (16/114). In addition to hyperventilation and SRB, their most striking feature was their marked ruddy complexion, which persisted as they completed growth to adulthood, with the occasional presence of dilated surface blood vessels reminiscent of telangiectasias (Fig. S3). Although the number of such animals was too low to significantly affect heterozygotes’ overall survival rate, we did observe a higher frequency of death events in this group (Fig. 2C, arrows), resulting in an estimated 5-month survival of 37.5% (6/16). Thus, similar to HHT1 mouse models, a single mutated *endoglin* allele leads to pathological conditions with limited penetrance, which is in line with the ‘second hit’ concept, in zebrafish as well.

### Vascular malformations in Endoglin-deficient fish

Because mutations affecting the *ENG* gene are responsible for HHT1 in humans, characterized by cerebral, pulmonary, hepatic or gastrointestinal AVMs, we examined the vascular architecture of dissected *eng^−/−^* brain, liver and intestine in *Tg(kdrl:GFP), Tg(flt1:Tomato)* transgenic background at 27[Supplementary-material sup1]dpf. In the optic tectum of *eng^−/−^* fish, enlarged and tortuous veins (GFP^+^, Tomato^−^) were frequently observed (5/11) and, more rarely, major arteries (GFP^+^, Tomato^+^) directly connected to veins (Fig. S4A′). Brain hemorrhages were also found in association with venous malformations (3/11) (Fig. S4A″). Imaging of the intestine occasionally revealed the presence of enlarged veins and abnormally organized arteries compared to those of siblings. The capillary bed also appeared locally affected, with an obvious decrease in density (Fig. S4B). Consistent with the occurrence of AVMs in zebrafish intestinal tract, we observed intestinal hemorrhages in older *eng^−/−^* fish (Fig. S5). In the liver, instead of a regular tree-like organization of arterial and venous networks, *eng^−/−^* fish displayed a honeycomb-like organization of blood vessels and lacked major hepatic veins (Fig. S4C). Therefore, at the juvenile stage and with the exception of the liver, *eng^−/−^* organs present vascular abnormalities mostly consisting of venous malformations highly reminiscent of HHT-associated vascular dysplastic lesions.

### Early detection of concomitant hypoxic and cardiac stress responses in Endoglin-deficient zebrafish

**Fig. 3.**
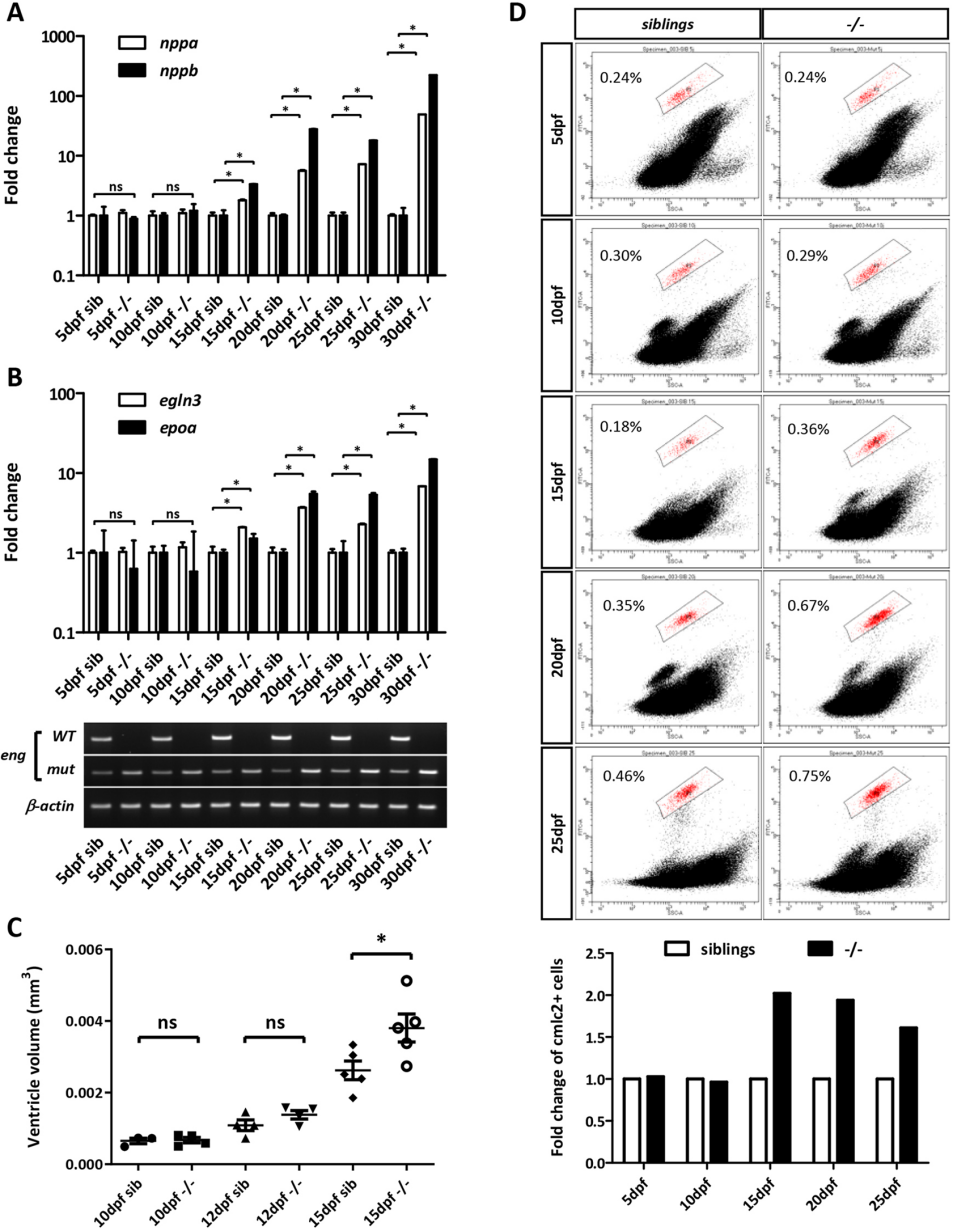
**Cardiac stress, ventricle enlargement and increased cardiomyocyte number correlate with hypoxia in Endoglin-deficient fish.** (A,B) RT-qPCR analysis of *nppa* and *nppb* (cardiac stress) (A), and *egln3* and *epoa* (hypoxia) (B), in 5, 10, 15, 20, 25 and 30 dpf sibling and *eng^−/−^* fish (RT-PCR genotyping is presented below graphs). Target gene expression values were normalized to *rpl13a*. 5 dpf and 10 dpf, *n*=15; 15 dpf, *n*=8; 20 dpf, *n*=5; 25 dpf, *n*=4; 30 dpf, *n*=3. Data are mean±s.e.m. of technical replicates. *nppa*: 15 dpf, 20 dpf, 25 dpf and 30 dpf, *P=0.05. *nppb*: 15 dpf, *P=0.05; 20 dpf, *P=0.0383; 25 dpf and 30 dpf, *P=0.05. *egln3*: 15 dpf and 20 dpf, *P=0.05; 25 dpf, *P=0.03831; 30 dpf, *P=0.05. *epoa*: 15 dpf, 20 dpf, 25 dpf and 30 dpf, *P=0.05. ns, not significant (one-tailed Mann–Whitney test). dfp, days postfertilization. (C) Analysis of ventricle volume in 10, 12 and 15 dpf sibling and *eng^−/−^* fish in *Tg(cmlc2:GFP)* background (*n*=5). Ventricle volume is estimated by the simplified ellipsoid volume calculation formula *V*=0.523(width in mm)^2^(length in mm). *P=0.0159; ns, not significant (one-tailed Mann–Whitney test). (D) Flow cytometry analysis of cardiomyocyte (*cmlc2:GFP*^+^) fraction in whole organism at 5, 10, 15, 20 and 25 dpf. Cell suspensions at 5, 10, 15, 20 and 25 dpf were prepared from pools of 26, 18, nine, seven and seven *eng^−/−^* fish or siblings, respectively. Bottom graph represents fold change in *cmlc2:GFP*^+^ cells in *eng^−/−^* fish relative to siblings. Data are representative of three independent experiments.

Because hypoxia is a potent driver of cardiac remodeling involving cardiomyocyte hypertrophy in mammals and both proliferation and hypertrophy in zebrafish (Jopling et al., 2012; Ostadal and Kolar, 2007; Sun et al., 2009), and because hyperventilation and SRB are hallmarks of hypoxia in fish, we reasoned that it might trigger heart failure in *eng^−/^*^−^ zebrafish. We monitored the expression of cardiac stress markers, i.e. atrial- and brain-type natriuretic peptides *nppa* and *nppb*, respectively, to pinpoint the onset of pathological cardiac remodeling in *eng^−/^*^−^ zebrafish. RT-qPCR analysis revealed that, by 15[Supplementary-material sup1]dpf, *eng^−/^*^−^ zebrafish exhibited 1.8- and 3.3-fold induction of *nppa* and *nppb*, respectively. These differences in expression between *eng^−/^*^−^ zebrafish and siblings increased to a 49- and 223-fold change in *nppa* and *nppb*, respectively, by 30[Supplementary-material sup1]dpf, consistent with the gradual enlargement of the *eng^−/^*^−^ heart region over time (Fig. 3A). We also analyzed the expression of hypoxia-responsive genes *egln3* (prolyl hydroxylase 3) and *epoa* (erythropoietin). In this manner, we found a 2.1-fold increase in expression for *egln3*, and 1.5-fold increase in expression for *epoa*, in 15[Supplementary-material sup1]dpf *eng^−/−^* fish compared to siblings. By 30 dpf, a 6.8- and 14.9-fold increase in *egln3* and *epoa*, respectively, were observed (Fig. 3B). To verify that increased natriuretic peptide expression reflected the onset of cardiomegaly in *eng^−/−^* zebrafish, we measured the ventricle volume of *eng^−/−^* zebrafish and siblings at 10, 12 and 15[Supplementary-material sup1]dpf. Although this volume was similar between *eng^−/^*^−^ zebrafish and siblings at both 10[Supplementary-material sup1]dpf and 12[Supplementary-material sup1]dpf, it appeared significantly increased in mutants at 15[Supplementary-material sup1]dpf (Fig. 3C). Histological sections from 10, 12, 15 and 20[Supplementary-material sup1]dpf *eng^−/−^* zebrafish and siblings confirmed these data. Interestingly, we did not observe any noticeable structural anomalies in *eng^−/−^* hearts (see Fig. 6A). Furthermore, we found that, by 20[Supplementary-material sup1]dpf, although the hearts of *eng^−/−^*zebrafish were significantly larger than those of their siblings, the thickness of the ventricular wall increased along with heart size, resulting in a ventricular wall thickness/heart size ratio similar to that of siblings (see Fig. 6B). This indicated that *eng^−/−^* heart enlargement is not due to dilatation and the associated thinning of the ventricle wall, but instead that cardiomegaly is the source of this phenotype. Finally, to define how the heart would specifically respond to hypoxic cues in Endoglin-deficient fish, we performed fluorescence-activated cell sorting (FACS) analysis to monitor changes in cardiomyocyte abundance. Analysis of *cmlc2:GFP*^+^ cardiomyocytes in cell suspensions from whole fish revealed a recurrent increase in GFP^+^/total cell ratio in 15[Supplementary-material sup1]dpf and older *eng^−/^*^−^ zebrafish, indicating that *eng^−/−^* hearts contained more cardiomyocytes than sibling hearts (Fig. 3D). Altogether, these data showed that Endoglin deficiency does not impact early heart development but instead results in cardiomegaly at later stages, leading eventually to terminal heart failure.

### Hypochromic anemia is not the primary trigger of heart failure in Endoglin-deficient fish

As anemic mutants such as *riesling* (also known as *sptb*) (Liao et al., 2000), *merlot*/*chablis* (also known as *epb41b*) (Shafizadeh et al., 2002) or *retsina* (also known as *slc4a1a*) (Paw et al., 2003) develop cardiomegaly as a result of cardiac compensation for oxygen transport, we sought to define whether hypochromic anemia in *eng^−/^*^−^ fish could account for hypoxia detected by 15[Supplementary-material sup1]dpf. We evaluated hemoglobin content using o-dianisidine at 3, 5, 10 and 15[Supplementary-material sup1]dpf. We did not observe any reduction in staining intensity in *eng^−/^*^−^ zebrafish compared with that in siblings at any given time point. Conversely, we noticed a trend towards enhanced staining in the *eng^−/^*^−^ group at 15[Supplementary-material sup1]dpf, which correlated with increased *epoa* expression at this point (Fig. S7). Consistent with this, large amounts of erythrocytes filling *eng^−/−^* heart chambers were observed at 15 and 20[Supplementary-material sup1]dpf (Fig. S6A), and blood smears from 25 and 30[Supplementary-material sup1]dpf *eng^−/^*^−^ fish revealed large numbers of erythrocytes, most with immature shape and abnormal staining (Fig. S8A). Furthermore, analysis of 30[Supplementary-material sup1]dpf *eng^−/^*^−^ kidney, the fish definitive hematopoiesis organ, revealed increased cellularity, indicative of reactive erythropoiesis (Fig. S8B). Lastly, adult surviving *eng^−/^*^−^ zebrafish displayed increased hematocrit compared to that of wild-type adult fish (Fig. S8C). These results indicated that loss of Endoglin results in enhanced erythropoiesis, most likely caused by increased *epoa* expression in response to hypoxia. They also showed that hypochromic anemia is not the primary cause of hypoxia but rather a late secondary acquired feature of Endoglin deficiency.

### Endoglin-deficient zebrafish exhibit defects in gill blood vessel development

**Fig. 4.**
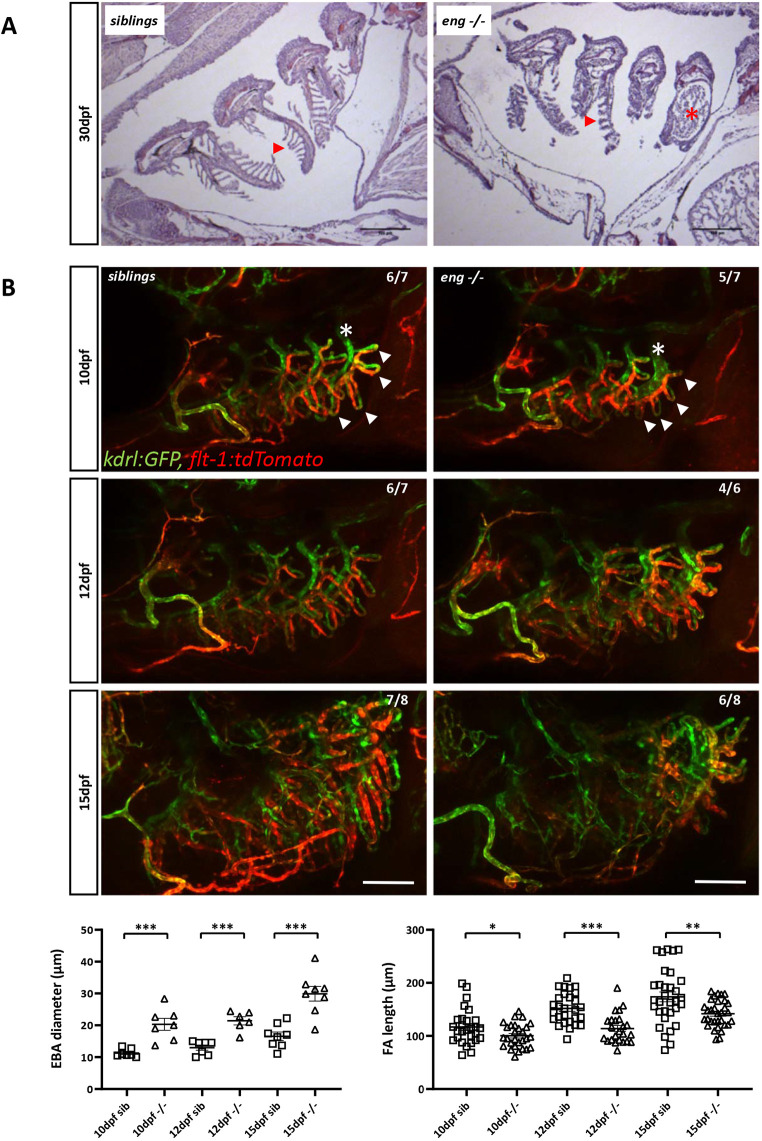
**Abnormal branchial vascular development in Endoglin-deficient fish underlies structural impairment of gills.** (A) H&E-stained histological sections of 30 dpf sibling and *eng^−/−^* gills. Note the poorly developed lamellae (arrowheads) and enlarged artery (asterisk) on branchial arch 4 (AA6) in *eng^−/−^* fish gills. Scale bars: 100 µm. (B) Top three rows: kinetic analysis of gill vascular development in 10, 12 and 15 dpf sibling and *eng^−/−^* fish in *Tg(kdrl:GFP), Tg(flt1:tdtomato*) background. Note the reduced length of afferent filamental artery (GFP^+^, Tomato^high^) and efferent filamental artery (GFP^+^, Tomato^low/−^) (arrowheads) and enlarged efferent branchial artery) (asterisks) as early as 10 dpf. Note the overall loss of *flt1* signal in 15 dpf *eng^−/−^* fish. Scale bars: 100 µm. Bottom row: graphical representation of sibling and *eng^−/−^* fish branchial efferent artery (EFA) diameter (left) and filamental artery (FA) length (right) at 10, 12 and 15 dpf. EFA diameter and FA length were measured on AA6. EFA diameter was measured at the most dorsal FA level. FAs (five per fish) were measured starting from the most dorsal FA. Data are presented as individual values and mean±s.e.m. EFA diameter: siblings (sib) versus −/−, ***P=0.0003, ***P=0.0006 and ***P=0.0003 at 10, 12 and 15 dpf, respectively. FA length: sib versus −/−, *P=0.037, ***P<0.0001 and **P=0.0026 at 10, 12 and 15 dpf, respectively. One-tailed Mann–Whitney test.

Because anemia did not appear to be at the source of hypoxia in *eng^−/−^* zebrafish, we hypothesized that this might reflect respiratory issues. Analysis of histological sections from 30[Supplementary-material sup1]dpf fish revealed that *eng^−/−^* gills were structurally abnormal. Lamellae were unusually short and crooked, and blood vessels were markedly enlarged, notably in the fourth branchial arch (AA6) (Fig. 4A). We thus compared *eng^−/−^* zebrafish and sibling gill vascular architecture at 10, 12 and 15[Supplementary-material sup1]dpf in *Tg(kdrl:GFP), Tg(flt1:Tomato)* transgenic background (Fig. 4B). Although gill vasculature is an arterioarterial system (Olson, 2002), Flt1 arterial marker was found mostly associated with afferent branches, whereas *kdrl* promoter appeared evenly active in both afferent and efferent parts of the gill vasculature. As early as 10[Supplementary-material sup1]dpf, we observed that vascular loops (afferent/efferent) were shorter in mutants (Fig. 4B, arrowheads) and that the efferent artery of the fourth branchial arch was substantially dilated (Fig. 4B, asterisks). These differences persisted at later stages, and, by 15[Supplementary-material sup1]dpf, although pan-vascular endothelium *kdrl* reporter expression remained essentially unchanged, we observed a striking loss of *flt1* reporter activity in the gill afferent arterial network, suggesting that arterial identity might be affected. Finally, to ascertain whether Endoglin played a direct role in gill formation, we analyzed *endoglin* expression by whole-mount *in situ* hybridization in wild-type fish. *endoglin* transcripts were detected in the developing gills of 10[Supplementary-material sup1]dpf larvae in a pattern that followed branchial arches and also in the paired anterior dorsal aortas. Expression increased and spread out by 12 and 15[Supplementary-material sup1]dpf, indicating that *endoglin* expression was not restricted to the major arteries but was also expressed in filamental arteries and lamellae (Fig. S9A,B). Notably, *endoglin* expression was absent in the heart at these stages, indicating that heart failure in *eng^−/^*^−^ zebrafish is indirect. These data indicated that, in *eng^−/−^* zebrafish, gill vascular development is defective, which manifests as hypoxemia-associated cardiomegaly and resulting heart failure.

### Induction of hypoxia in wild-type zebrafish does not recapitulate Endoglin deficiency cardiac phenotype

**Fig. 5.**
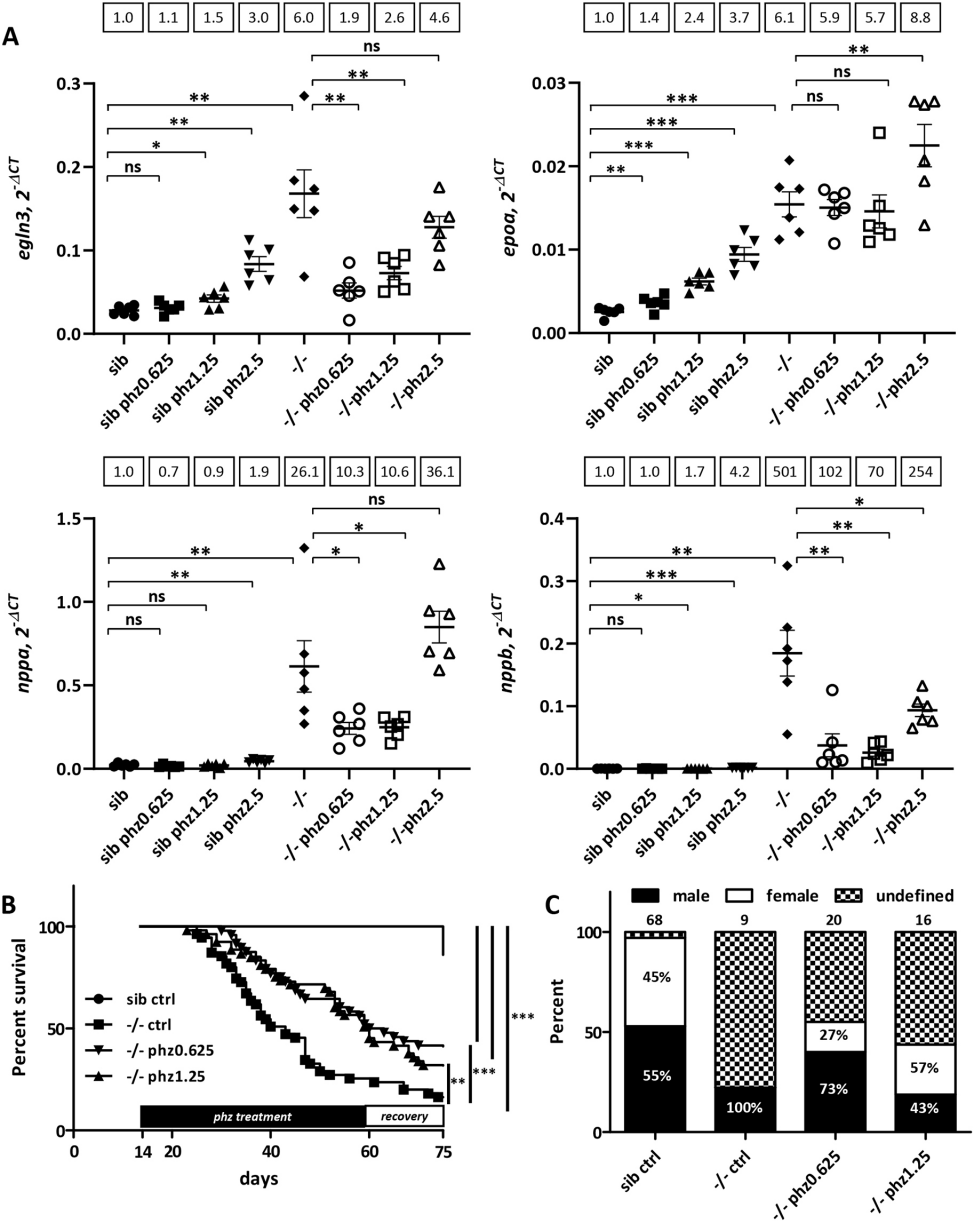
**Phenylhydrazine treatment alleviates pathological conditions induced by Endoglin deficiency in zebrafish.** (A) RT-qPCR analysis of *egln3*, *epoa* and *nppa* and *nppb* expression in 27 dpf sibling and *eng^−/−^* fish untreated or treated with 0.625, 1.25 and 2.5 µg/ml phenylhydrazine (phz). Target gene expression is represented as 2^−ΔCT^ using *rpl13a* as reference. Samples (*n*=6) are pools of four fish. Data are presented as individual values and mean±s.e.m. Mean fold change using sib as reference is indicated above graphs. *egln3*: sib versus sib phz 0.625 µg/ml (phz0.625), *P*=0.1178; sib versus sib phz 1.25 µg/ml (phz1.25), *P=0.0104; sib versus sib phz 2.5 µg/ml (phz2.5), **P=0.0019; sib control (ctrl) versus −/− ctrl, **P=0.0037; −/− ctrl versus −/− phz0.625, **P=0.005; −/− ctrl versus −/− phz1.25, *P*=0.0103; −/− ctrl versus −/− phz2.5, *P*=0.0814. *epoa*: sib versus sib phz0.625, **P=0.0068; sib versus sib phz1.25, ***P<0.0001; sib versus sib phz2.5, ***P=0.0003; sib ctrl versus −/− ctrl, ***P=0.0003; −/− ctrl versus −/− phz0.625, *P*=0.1573; −/− ctrl versus −/− phz1.25, *P*=0.1489; −/− ctrl versus −/− phz2.5, **P=0.0094. *nppa*: sib versus sib phz0.625, *P*=0.0525; sib versus sib phz1.25, *P*=0.1850; sib versus sib phz2.5, **P=0.0014; sib ctrl versus −/− ctrl, **P=0.0045; −/− ctrl versus −/− phz0.625, *P=0.0204; −/− ctrl versus −/− phz1.25, *P=0.0206; −/− ctrl versus −/− phz2.5, *P*=0.0675. *nppb*: sib versus sib phz0.625, *P*=0.3146; sib versus sib phz1.25, *P=0.0417; sib versus sib phz2.5, ***P=0.0005; sib ctrl versus −/− ctrl, **P=0.0032; −/− ctrl versus −/− phz0.625, **P=0.0043; −/− ctrl versus −/− phz1.25, **P=0.0042; −/− ctrl versus −/− phz2.5, *P=0.0259. ns, not significant. Shown are false discovery rate (FDR)-adjusted *P*-values from Brown–Forsythe test and Welch's test, and two-stage linear set-up procedure of Benjamini, Krieger and Yekutieli posthoc test for multiple comparisons after assessment of normal distribution and equal s.d. (B) Phz treatment enhances *eng^−/−^* survival. Kaplan–Meier representation of the survival of sibling and *eng^−/−^* fish non-treated (ctrl) or treated with phz at 0.625 or 1.25 µg/ml. sib versus −/− ctrl or −/−phz1.25 or −/−phz0.625, ***P<0.0001; −/− versus −/− phz1.25, **P=0.0039; −/− versus −/− phz0.625, ***P=0.0009 [log-rank (Mantel–Cox) test]. sib ctrl, *n*=66; −/− ctrl, *n*=55; −/−phz1.25, *n*=53; −/−phz0.625, *n*=48. (C) Influence of phz treatment on sex ratio in individuals aged 2.5 months and above. Graph represents the percentage of fish reaching adulthood. Analyzed fish numbers are indicated above bars. Male and female values are indicated inside bars.

To define how hypoxia specifically contributed to heart failure in *eng^−/−^* zebrafish, we treated wild-type fish with the hemolytic agent phz, providing a versatile model of hypoxia. To match the onset of hypoxia, phz was applied every other day from 14 dpf, and hypoxic and cardiac stress response was assessed in 29 dpf fish. Hypoxia markers gradually increased with phz concentration and plateaued at 5 µg/ml (Fig. S10A, top row). Although cardiac stress markers followed a similar trend, we only observed a significant increase following acute phz exposure (Fig. S10A, bottom row), suggesting that, in contrast to in mammals, *nppa* and *nppb* are, at best, weak hypoxia-responsive genes in zebrafish (Arjamaa and Nikinmaa, 2011). In 5 µg/ml phz-treated fish, *nppa* expression increased by 2.4-fold and *nppb* expression by 6.6-fold, whereas *egln3* and *epoa* expression increased by 3.2-fold and 2.8-fold, respectively. Thus, although hypoxic responses in phz-treated wild-type and *eng^−/^*^−^ zebrafish were in the same range of magnitude, cardiac stress in phz-treated fish appeared negligible with regard to that in *eng^−/−^* fish (see Fig. 3A,B and Fig. 5A for comparison). Consistent with this, 5 µg/ml phz-treated fish showed only mild enlargement of the heart area compared to that in age-matched *eng^−/^*^−^ fish (Fig. 2E; Fig. S10B). Phz treatment had no effect on survival up to 30 dpf. Prolonged treatment affected survival in a dose-dependent manner, but the effects on fish survival, compared to that of *eng^−/−^* fish, were marginal [survival at 75 dpf: phz 1.25 µg/ml, 91.9%; phz 2.5 µg/ml, 90.1%; phz 5 µg/ml, 75.8%; versus *eng^−/−^*, 38.5% (survival from Fig. 2C adjusted for comparison)] (Fig. S10C). Furthermore, analysis of sex ratio at 3 months, after 1 month of recovery, did not reveal altered male/female ratio, indicating that the male sex bias effect in *eng^−/−^* fish was not directly induced by hypoxia (Fig. S10D). Collectively, these results showed that chronic hypoxia is not sufficient to recapitulate *eng^−/−^* cardiac phenotype.

### Hypoxia-induced erythropoiesis is detrimental to Endoglin-deficient fish

Because phz induces hypoxia via its hemolytic activity, whereas, in *endoglin* mutant zebrafish, hematocrit/blood viscosity is increased as a result of hypoxia, we assessed whether increased hematocrit would contribute to heart failure in Endoglin-deficient fish. We treated *eng^−/^*^−^ zebrafish and siblings with phz at concentrations (0.625, 1.25 and 2.5 µg/ml) that showed minimal effect on hypoxia and cardiac stress in wild-type fish (Fig. S10A). These low phz concentrations showed no overt effect on siblings compared to untreated counterparts, and *eng^−/^*^−^ zebrafish treated with phz appeared healthier with less prominent cardiomegaly than untreated *eng^−/−^* zebrafish (not shown). We measured hypoxia and cardiac stress in these different settings at 27[Supplementary-material sup1]dpf (Fig. 5A). Similar to the results shown in Fig. 3A and B, untreated *eng^−/^*^−^ zebrafish exhibited a 6.0-fold increase in *egln3* expression, 6.1-fold increase in *epoa* expression, 26.1-fold increase in *nppa* expression and 501-fold increase in *nppb* expression compared to that in their untreated siblings. We found that the lowest phz concentrations (0.625 and 1.25 µg/ml) had no or modest effect on *egln3*, *epoa*, *nppa* and *nppb* expression in siblings, whereas, in *eng^−/^*^−^ fish, these phz concentrations reduced *nppa* and *nppb* induction of expression (mean fold increase compared to that in non-treated siblings group for *eng^−/^*^−^ fish treated with phz at 0.625 and 1.25 µg/ml: *nppa*, 10.6; *nppb*, 102 and 70, respectively). Low phz concentrations did not increase the expression of hypoxia markers in *eng^−/^*^−^ and even reduced it if considering only *egln3* (mean fold increase compared to that in non-treated siblings for *eng^−/−^* versus *eng^−/^*^−^ treated with phz at 0.625 and 1.25 µg/ml: *epoa*, 6.1 versus 5.9 and 5.7, respectively; *egln3*, 6.0 versus 1.9 and 2.6, respectively), suggesting that heart failure would further worsen oxygenation. To gain more insight into the effect of phz treatment on *eng^−/−^* heart, we produced histological sections from 27 dpf siblings, *eng^−/−^* fish and *eng^−/−^* fish treated with 0.625 and 1.25 µg/ml phz. Analysis revealed that phz markedly reduced ventricle size and preserved the internal structure of *eng^−/−^* heart (Fig. S11A,B). We, thus, evaluated whether phz treatment would provide long-term benefit to *eng^−/^*^−^ fish health. Fish were treated with either 0.625 or 1.25 µg/ml phz every other day from 14 dpf and allowed to recover from 60 dpf to 75 dpf. As shown in Fig. 2C, *eng^−/^*^−^ fish survival was severely impaired compared to that of siblings, with a 16.4% survival at 75 dpf (median survival, 43 days). By contrast, the survival of phz-treated *eng^−/^*^−^ fish was markedly enhanced, reaching 41.7% for the phz 0.625 µg/ml group and 32.1% for the phz 1.25 µg/ml group (median survival of phz 0.625 µg/ml group and phz 1.25 µg/ml group, 61.5 and 60 days, respectively) (Fig. 5B). Further highlighting the attenuating effect of phz on the *eng^−/^*^−^ cardiac phenotype, treatment allowed higher *eng^−/^*^−^ fractions to reach adulthood and corrected the sex bias toward males (Fig. 5C). Altogether, these results showed that hypoxia-induced erythropoiesis is a major contributor to heart failure in Endoglin-deficient zebrafish.

### Embryonic circulatory pattern defects and juvenile heart failure in Endoglin-deficient fish are rescued by endothelial-specific transgenic expression of either long or short isoform Endoglin

**Fig. 6.**
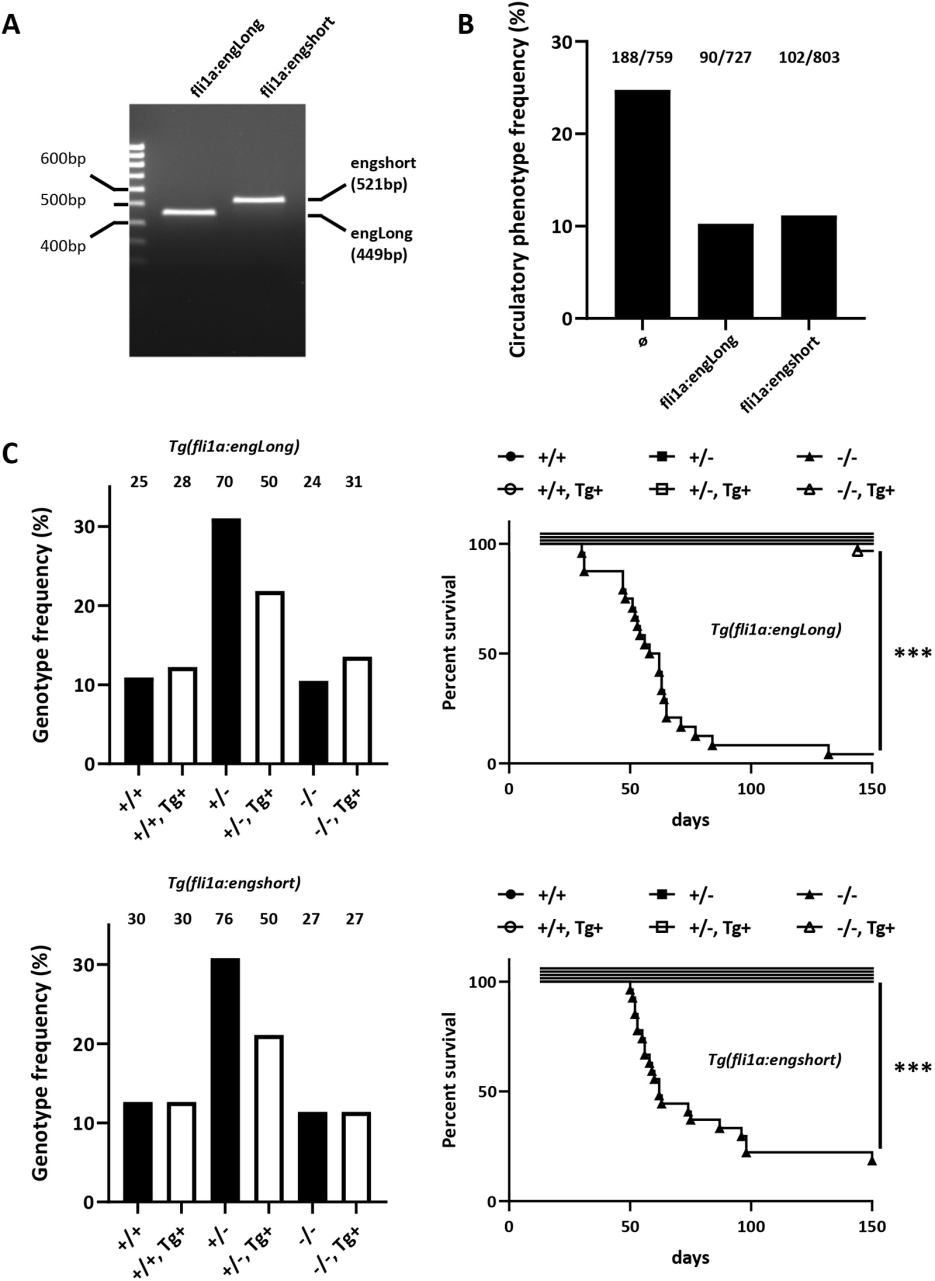
**Both long and short Endoglin isoforms rescue *eng^−/−^* heart failure.** (A) PCR genotyping example of established *fli1a:engLong* and *fli1a:engshort* transgenic lines. Amplicon size difference is due to exon 13 inclusion in *endoglin* short coding transcript. (B) Mutant phenotype frequency in clutches from, *eng^+/−^, Tg(fli1a:engLong)* and *eng^+/−^, Tg(fli1a:engshort)* with *eng^+/−^* fish at 3 dpf. Total number of mutants over total number of fish analyzed is indicated above bars. (C) Genotype distribution frequency and associated survival in 5-month-old fish from crosses between *eng^+/−^* and *eng^+/−^, Tg(fli1a:engLong)* or *eng^+/−^, Tg(fli1a:engshort)*. Left column shows even transgene distribution among the different genotypes. Fish numbers are indicated above bars. Right column shows Kaplan–Meier representation of survival according to genotype and transgenic status. −/− versus −/−*, Tg(fli1a:engLong)* or *Tg(fli1a:engshort)*, ***P<0.0001 [log-rank (Mantel–Cox) test].

In order to define whether heart failure in *eng^−^*^/−^ fish relies on the lack of a specific Endoglin isoform, we produced transgenic lines expressing either long or short isoform Endoglin under the regulation of the EC-specific zebrafish *fli1a* promoter/enhancer. We selected transgenic lines carrying a single copy of Endoglin long or short isoform transgenes (Fig. 6A). Although, in *eng^+/−^* incrosses, mutant circulatory phenotype at 3 dpf was observed at a 25% frequency, it dropped to ∼12.5% in crosses of *eng^+/−^, Tg(fli1a:engLong)* or *eng^+/−^, Tg(fli1a:engshort)* with *eng^+/−^* fish (Fig. 6B), indicating that both Endoglin isoforms individually rescue embryonic *eng^−/−^* circulatory phenotype. Then, clutches obtained from crosses of *eng^+/−^, Tg(fli1a:engLong)* and *eng^+/−^, Tg(fli1a:engshort)* with *eng^+/−^* fish were raised to adulthood (5 months), and individual fish including those that died in the course of experiments were genotyped to define their mutation and transgene status and associated survival. As expected, transgenes distributed evenly across the different genotypes (Fig. 6C, left column). As already shown in Fig. 2C and Fig. 5B, *eng^−/−^* zebrafish survival was greatly impaired, but was fully restored by re-expression of either Endoglin long or short isoform (Fig. 6C, right column). These data, thus, indicated that Endoglin cytosolic domain has no important function with regard to embryonic circulatory or heart failure phenotype and that, in zebrafish, specific protein interactions involving Endoglin cytosolic domains are physiologically dispensable.

## DISCUSSION

In the present work, we described the generation of a CRISPR/Cas9 *endoglin* zebrafish mutant and the consequences of Endoglin deficiency in postembryonic stages. We showed that mutant zebrafish die from congestive heart failure accompanying chronic hypoxia, which likely results from gill dysfunction. We showed that the health and survival of Endoglin-deficient fish can be strongly enhanced by treatment with the hemolytic agent phz, demonstrating that increased hematocrit/blood viscosity induced by hypoxia is a main driver of heart failure in these fish. Finally, we found that, individually re-expressed in Endoglin-deficient fish, the phylogenetically conserved Endoglin long and short isoforms similarly rescue heart failure.

Our data provide supporting evidence that the *endoglin* mutant phenotypes described here truly reflect Endoglin deficiency. It is thus surprising that the previous *endoglin* mutant described by Sugden et al. (2017) does not suffer from heart failure even though both mutants display the same embryonic phenotype. Genetic compensation particularly stands out to explain this discrepancy. This phenomenon, also called transcriptional adaptation, triggers the upregulation of genes with sequence similarities to a given mutated mRNA and is specifically induced by mutations inducing premature termination codon (PTC) and ensuing mRNA decay (El-Brolosy et al., 2019). In the previous *endoglin* mutant, the indel results in a PTC and strong NMD. In our mutant, by contrast, the indel destroys the *endoglin* start codon and Kozak sequence to convert *endoglin* messenger into a non-coding RNA, which should not trigger genetic compensation. We, nevertheless, observed a slight decrease in *endoglin* mRNA abundance in mutants, which could reflect stability differences between wild-type and mutated mRNA or Endoglin self-regulation at the transcription level.

The zebrafish *endoglin* locus expresses two distinct transcripts via the use of an alternative exon, introducing an early stop codon and resulting in two Endoglin proteins with unique cytosolic sequences. Similar isoforms are also expressed in mammals (Bellón et al., 1993). However, in mammals, endoglin short isoform is produced by an intron retention mechanism involving splicing factor 2 (ASF/SF2) (Blanco and Bernabeu, 2011). Zebrafish Endoglin overall similarity with mammal orthologs is globally low, but the cytosolic region of the long isoform is remarkably homologous to that of mammals, which interacts with proteins such as GIPC, β-arrestin2 and NOS3 (Lee and Blobe, 2007; Lee et al., 2008; Toporsian et al., 2005). The zebrafish Endoglin short isoform cytosolic domain adds to the wide diversity of sequences of endoglin short isoform cytosolic domains found in mammals (Blanco and Bernabeu, 2011), and suggests that this domain has no protein-binding function. Conservation underscores that both Endoglin isoforms should be essential, although the short isoform seems to be absent in reptiles and birds. In mammals, endoglin short isoform is induced in senescent ECs, and transgenic mice overexpressing this isoform are hypertensive and insensitive to the NO synthesis inhibitor L-NAME (Blanco et al., 2008). In this study, however, we found that Endoglin individual isoforms identically rescued *eng^−/−^* phenotypes, indicating that protein interactions with a specific Endoglin isoform cytosolic domain are not physiologically crucial in zebrafish. In mammals, a soluble form (S-endoglin) is also produced by cleavage of the ectodomain by MMPs and can exert paracrine or remote activity using exosomes as vehicle (Ermini et al., 2017). High S-endoglin levels correlate with preeclampsia and induce hypertension by inhibiting the TGF-β–NOS axis (Venkatesha et al., 2006). Further work will be necessary to establish whether S-Endoglin is produced in zebrafish, but sequence analysis indicates that ectodomain Glu-Leu residues essential for MMP14 cleavage in mammals are conserved (Hawinkels et al., 2010).

In zebrafish, in contrast to in mammals, Endoglin participation in ALK1 signaling seems highly questionable. Expression patterns overlap marginally during development, and differences in lethality timing between *alk1* and *endoglin* mutants (7-10 dpf versus 30 dpf, respectively) argue against function in a shared pathway (Roman et al., 2002). Zebrafish deficient for ALK1 or for both Bmp10 and Bmp10-like exhibit enlarged cranial blood vessels as a result of impaired EC ability to migrate against blood flow (Corti et al., 2011; Laux et al., 2013; Rochon et al., 2016; Roman et al., 2002). This phenotype is absent in *endoglin* mutants and morphants (Fig. S12). Endoglin, while acting as a co-receptor, might be somehow dispensable for BMP10 activation of ALK1 signaling. In static human umbilical vein EC cultures, *ENG* knockdown does not prevent ALK1 signaling in response to physiological concentrations of recombinant BMP9, whereas, in the same cells in fluid shear stress condition, endoglin is required for ALK1 response to very-low BMP9/10 concentrations (Baeyens et al., 2016). Serum concentrations of BMP9 were reported to greatly exceed its half-maximal effective concentration (David et al., 2007, 2008), but this notion is not really experimentally supported *in vivo*. Indeed, inducible EC-specific *Eng* knockout results in the formation of AVMs in the retina, similarly to ALK1 or SMAD4 deficiency or BMP9/10 inhibition (Singh et al., 2020; Baeyens et al., 2016; Crist et al., 2018). Moreover, *endoglin* mutant embryonic phenotype (this study; Sugden et al., 2017) is absent in *alk1*-deficient zebrafish, which instead exhibit a limited trunk circulation. ALK5 seems not to be an alternative candidate receptor because both *alk5a* and *alk5b* (also known as *tgfbr1a* and *tgfbr1b*, respectively) are absent in axial blood vessels by the time Endoglin deficiency induces changes in blood flow pattern (Park et al., 2008). Data, in mammals, suggest that endoglin can act outside of BMP/TGF-β signaling. Endoglin was indeed found to interact with VEGF-R2 and promote its signaling in response to VEGF (Tian et al., 2018). Further investigation will be required to define whether, in zebrafish, phenotypes induced by Endoglin deficiency can be related to this specific signaling. Interestingly though, ALK1, BMP9/BMP10 and endoglin knockout in adult mice similarly result in high-output heart failure, as does Bmp10 deficiency in zebrafish (Bouvard et al., 2022; Capasso et al., 2020; Morine et al., 2017; Tual-Chalot et al., 2020). Thus, ALK1–Endoglin interaction might still be required at later stages in zebrafish.

Our results demonstrate that zebrafish Endoglin deficiency elicits a chronic hypoxic response, which results in increased erythropoiesis and hypochromic anemia in late stages. In normoxia, HIFs (HIF-1α/HIF-2α) are hydroxylated by prolyl hydroxylases (PHDs), which use ferrous iron ions (Fe^2+^) as co-factor. Hydroxylated HIFs are recognized by the Von Hippel Lindau protein (pVHL) component of E3 ubiquitin ligase complex, leading to HIF polyubiquitylation and their degradation by the proteasome. Conversely, hypoxia hampers PHD activity leading to HIF stabilization, nuclear translocation and association with HIF-1β/ARNT to form a transcription complex that regulates specific sets of genes (Ivan and Kaelin, 2017). Iron deficiency leads, eventually, to anemia due to the essential contribution of iron to the heme complex, essential to hemoglobin for oxygen transport. An iron-free diet represses the iron reabsorption inhibitor hepcidin via an HIF-dependent transcriptional repression mechanism (Peyssonnaux et al., 2007). In keeping with this, hypoxia mimetics desferrioxamine and cobalt or nickel chloride inhibit PHDs by interfering with ferrous iron (Muñoz-Sánchez and Chánez-Cárdenas, 2019). Endoglin-deficient fish exhibit features highly reminiscent of zebrafish *vhl* mutants, which model Chuvash syndrome. These mutations abolish Vhl association with Hif factors and lead to the constitutive activation of Hif-dependent pathways in normoxia. Zebrafish *vhl* mutants exhibit exacerbated erythropoiesis in response to Epo overexpression, which results in a form of polycythemia with immature and hypochromic characteristics. These fish also exhibit a hyperventilation phenotype, which stems from the direct action of Epo on respiratory neurons, and develop congestive heart failure, edema and die by 11 dpf (van Rooijen et al., 2009). In mouse, liver-specific deletion of VHL results in HIF-mediated hepcidin repression and EPO induction, leading to excessive (polycythemia) microcytic and hypochromic erythropoiesis. Thus, despite converging mechanisms set to mobilize iron and red blood cell production, conditions of chronic HIF stabilization end up invariably in an imbalance between EPO and iron levels (Peyssonnaux et al., 2007).

Chronic hypoxia induces cardiomegaly in both fish and mammals, but unlike mammal cardiomyocytes, which undergo size increment (hypertrophy), fish cardiomyocytes are able to re-enter the cell cycle after dedifferentiation (Jopling et al., 2010). Our data, although indirectly, show that cardiomyocyte proliferation is enhanced in Endoglin-deficient fish and coincides with hypoxia. Recent studies have shed light on how HIF activation induces cardiomegaly through cardiomyocyte hypertrophy and cardiac remodeling. This involves hypoxia-inducible mitogen factor (HIMF), a non-canonical ligand of calcium-sensing receptor (CASR), which controls cardiomyocyte hypertrophy by activating HIF, CaN-NFAT and MAPK pathways (Kumar et al., 2018; Zeng et al., 2017). Whether these pathways could also be involved in zebrafish is still awaiting clarification, but HIMF potential orthologs are, to date, absent in zebrafish databanks.

In contrast to in *vhl* mutants, hypoxic response in *endoglin* mutants is gradually acquired. This indicates that Endoglin does not control Hif activation. Our data instead strongly argue in favor of a faulty respiratory system in Endoglin-deficient zebrafish. Fish extract water-dissolved oxygen through their gills, a highly complex respiratory organ composed of multiple functional units called lamellae. Lamellae are thin flat vascular sinusoids of endothelial and non-endothelial pillar cells ensheathed by a layer of pavement epithelial cells (Olson, 2002). In zebrafish, gills lamellae form at ∼12-14 dpf to reach their definitive adult morphology by ∼4 weeks (Rombough and Drader, 2009). Our findings that Endoglin is expressed during gill development and that Endoglin-deficient zebrafish exhibit structural and molecular alterations of the gill vasculature suggest that these defects directly impair lamellae development and gill function, resulting in hypoxemia. It is thus not surprising that mutant fish will start to decline by 30 dpf, which corresponds to the time when the gill normally reaches its fully operative shape in zebrafish, although variability in the extent of gill dysfunction may allow some mutant fish to survive longer.

Treating Endoglin-deficient zebrafish with the hemolytic agent phz has a strong impact on fish health. Enhanced survival clearly demonstrates that this prevents heart failure in *endoglin* mutants. A beneficial effect of phz has been demonstrated for hematocrit management originally in cases of polycythemia vera (Long, 1926) and later in secondary polycythemia (such as in chronic obstructive pulmonary disease), two pathologies associated with heart failure, when used in combination with pyrimethamine (Pengelly, 1969). Phz toxicity has caused its use to be abandoned for safer approaches such as phlebotomy. This, however, supports the notion that a form a polycythemia is taking place in Endoglin-deficient zebrafish that puts the heart at stake. Interestingly, in HHT, polycythemia is a complication of pulmonary AVM, which, by mixing arterial and venous blood (right-to-left shunting), results in hypoxemia, eventually triggering erythropoiesis (Cottin et al., 2007; McDonald et al., 2011). Thus, despite structural differences between fish and human cardiorespiratory systems, Endoglin deficiency appears to result in similar pathological situations.

Zebrafish has become a choice organism in functional screens of molecules of therapeutic interest. Hence, by reproducing important features of HHT, our *endoglin* mutant zebrafish will give us the opportunity to address the ‘second hit’ issue by screening for drugs able to induce symptoms at a higher frequency in heterozygotes, while homozygotes will serve to identify molecules able to rescue both embryonic and postembryonic phenotypes.

## MATERIALS AND METHODS

### Zebrafish lines, maintenance and ethics

All zebrafish (*Danio rerio*) lines were produced or maintained in AB genetic background. Transgenic lines used are as follows: *Tg(myl7:EGFP)*, referred to as *Tg(cmlc2:EGFP)* (provided by C. Jopling, Solutions de Génétique Fonctionnelle, Montpellier, France); *Tg(-0.8flt1:RFP)^hu5333^*, referred to as *Tg(flt1:Tomato)*; *Tg(kdrl:GFP)^la116^*; *Tg(-8.1gata1a:mRFP)^ko05^*, referred to as *gata1:RFP*. Embryos were staged according to Kimmel et al. (1995), and blood vessel nomenclature refers to Isogai et al. (2001). All experiments were performed in accordance with the 2010/63/EU Directive and the ARRIVE guidelines (Kilkenny et al., 2010). Procedures used in this study have been approved by Ethical Committee (#036) as part of an authorized project registered by the French Ministère de l'Enseignement Supérieur de la Recherche et de l'Innovation under APAFIS #23822-2020052717421507 v3 (E.L.). Animals were housed in a zebrafish facility registered under Agreement #A3417237.

### Total RNA extraction, semi-quantitative PCR and RT-qPCR analysis

Total RNA was extracted from staged embryos, sibling and *eng^−/−^* fish at specific ages as pool using Nucleospin RNA kit (Macherey-Nagel) or NucleoZOL (Macherey-Nagel) following the manufacturer's instructions. Equal amounts (500 ng or 1 µg) of total RNA were retrotranscribed using a High Capacity cDNA Reverse Transcription kit (Applied Biosystems) following the manufacturer's instruction. *endoglin* variants were amplified by semi-quantitative PCR using Phusion Hot Start II High-Fidelity DNA polymerase (Thermo Fisher Scientific) or HotGoldStar PCR mix (Eurogentec) using 50 ng total RNA equivalent reverse transcription reaction with *endoglin*-specific primers (engExon12fwd, 5′-CTGGGCATAGCGTTCGGAGGATT-3′; engExon10fwd, 5′-CCTGGGACCTCGAATGTGCTGTAA-3′; engExon15rev, 5′-CATGCTGCTGGTGGGTGTGCT-3′), and housekeeping gene *actb1* primers (bactinfwd, 5′-CCTGGAGAAGAGCTATGAGCTG-3′; bactinrev, 5′-ATGGGCCAGACTCATCGTACTC-3′) as control. The genotype of samples was verified by RT-PCR using engWTfwd, engMutfwd (see ‘CRISPR/Cas9 generation of *endoglin* mutant and genoyping’ section for sequences) and engExon5rev (5′-CGTTGGTGACGGATGTGACT-3′) according to the semi-quantitative PCR procedure described above. Quantitative PCR reactions were carried out using 1.25 ng total RNA equivalent reverse transcription reaction in sensiFAST SYBR No-Rox mix (Bioline) in the presence of 600 nM of each primer. Reactions were assembled in triplicates in 384-well plates using Labcyte Echo 525 Liquid Handler, and PCR was performed using the Roche LightCycler 480 available at Montpellier GenomiX's High-throughput qPCR facility. PCRs were conducted using the following primers: dreegln3fwd, 5′-TGGGAAAAAGCATTCGTGCG-3′; dreegln3rev, 5′-CGGCCATCAGCATTAGGGTT-3′; dreepoafwd, 5′-CCATTACGCCCCATCTGTGA-3′; dreepoarev, 5′-GTGACGTTCGTTGCAATGCT-3′; drenppafwd, 5′-GACACAGCTCTGACAGCAACA-3′; drenpparev, 5′-TCTACGGCTCTCTCTGATGCC-3′; drenppbfwd, 5′-TGTTTCGGGAGCAAACTGGA-3′; drenppbrev, 5′-GTTCTTCTTGGGACCTGAGC-3′; drerpl13afwd, 5′-CGCTATTGTGGCCAAGCAAG-3′; drerpl13arev, 5′-TCTTGCGGAGGAAAGCCAAA-3′; dreengfwd, 5′-AGACGGAGAACGGGACAGAA-3′; dreengrev, 5′-TCACCACAGACTTGTTCGCC-3′.

### 5′ and 3′ RACE experiments

For *endoglin* 5′ RACE, we used a template-switch strategy based on a previously described procedure (Pinto and Lindblad, 2010). Briefly, 36 h postfertilization (hpf) total RNA (1 µg) was retrotranscribed with either 5′-CATGCTGCTGGTGGGTGTGCT-3′ or 5′-CCATAAAGCACCGGTGAGCAGAA-3′ *endoglin*-specific reverse oligonucleotides (500 nM) using 10 U/µl RevertAid H Minus Reverse Transcriptase (Thermo Fisher Scientific) in 1× RevertAid H Minus Buffer supplemented with 2 U/µl RNAse OUT (Thermo Fisher Scientific), 1 mM dNTPs (Thermo Fisher Scientific), 2 mM MgCl_2_ at 50°C for 1 h. The template-switch reaction was then conducted in the presence of Template Switch oligonucleotide (1 µM), 3 mM MnCl_2_ and 4 U/µl RevertAid H Minus Reverse Transcriptase for 90 min at 42°C. Reverse transcriptase was finally inactivated by a 10 min at 70°C step. Then, using Template Switch RTs (50 ng total RNA equivalent) as template together with shortened U_sense oligonucleotide (5′-GTCGCACGGTCCATCGCAG-3′) and *endoglin*-specific oligonucleotides (5′-CCATAAAGCACCGGTGAGCAGAA-3′ and 5′-GTTTATCCTTTTGACGCCGCAGAG-3′) as primers, PCR reactions were performed using Phusion Hot Start II High-Fidelity DNA polymerase (Thermo Fisher Scientific) following the manufacturer's instructions. 3′ RACE was performed using a GeneRacer kit (Thermo Fisher Scientific) following the manufacturer's recommendations. Briefly, 36 hpf total RNA (1 µg) was used in reverse transcription reactions containing 2.5 µM GeneRacer Oligo dT primer and 10 U/µl SuperScript III RT. PCR reactions were then conducted as described above using *endoglin*-specific forward primer (5′-CCTGGGACCTCGAATGTGCTGTAA-3′) and GeneRacer 3′ primer. One percent of whole PCR was then used as template in nested PCR reactions using *endoglin*-specific forward primer (5′-CTGGGCATAGCGTTCGGAGGATT-3′) and GeneRacer 3′ Nested primer. PCR products from 5′ and 3′ RACE were then cloned blunt into pBSSK(−) vector and sequenced on both strands using T3 and T7 primers.

### Whole-mount *in situ* hybridization

Whole-mount *in situ* hybridization was performed essentially as previously described (Thisse and Thisse, 2008). Briefly, wild-type AB fish embryos or larvae were fixed overnight at 4°C in 4% paraformaldehyde (PFA), pH 9.5. Digoxygenin-labeled *endoglin* antisense riboprobe was synthesized from *endoglin* partial cDNA obtained from 5′ RACE (nucleotide 1 to 1454) cloned into pBSSK(−) vector. Samples were pre-hybridized overnight at 65°C, and hybridization was performed overnight at 65°C in the presence of 0.4 ng/µl *endoglin* antisense probe. Probe was detected using alkaline phosphatase-conjugated anti-digoxygenine Fab fragments (Roche) and NBT/BCIP reagent mix (Roche).

### CRISPR/Cas9 generation of *endoglin* mutant and genoyping

One nanoliter of a mixture composed of *endoglin*-targeting gRNA (TACGENE) and *Streptococcus pyogenes* Cas9-3NLS recombinant protein (TACGENE) (150 nM each) in 20 mM HEPES pH 7.5, 150 mM KCl was injected into four- to 16-cell-stage wild-type AB embryos. Fish were raised to adulthood, and indel carriers were screened by T7 Endonuclease I (T7EI) assay following PCR amplification from crude genomic DNA. T7EI^+^ samples were sequenced and analyzed using TIDE software. Selected founder F_0_ candidates were crossed with wild-type AB and progeny (F_1_) raised to adulthood. Indel carriers were identified using T7EI assay and direct sequencing of PCR products. A single *endoglin* mutant line was maintained for further analysis. F_1_, F_2_ or F_3_ mutation carriers were then crossed with transgenic reporter lines. Throughout this study, *endoglin* mutation was maintained in a heterozygous status to prevent the potential selection of individuals able to cope with Endoglin deficiency. Routine genotyping was performed on crude genomic DNA prepared from caudal fin clips or, in the case of survival experiments, pieces from dead larvae or juveniles using an allele-specific PCR strategy with primers (engWTfwd, 5′-ACAGACGAATCTACAGCCGACAT-3′; engMutfwd, 5′-AGAACAGACGAATCTACAGCCGAA-3′; engintron2rev, 5′-AGCATGTTTTAACAAGACGGCAG-3′) and HotGoldStar PCR mix (Eurogentec).

### Production of Endoglin-specific isoform transgenic rescue lines

A Tol2 transposon-based strategy was retained for the generation of transgenic fish lines. We used a modified version of pTLR vector (Peng et al., 2010), which carries SV40 (early) polyadenylation signal sequences retrieved from pEGFP-1 vector (Clontech) using NotI and AflII and cloned at NotI and SacII of pTLR Multi Cloning Site. *fli1a* promoter/enhancer was produced by PCR as previously described (Buchner et al., 2007) and re-amplified using 5′-CTCGAGGAGATCTCATCTTTGACCCATA-3′ and 5′-GTTTAAACGCGTCTGAATTAATTCCAGCC-3′ primers to introduce XhoI and PmeI restriction sites (underlined), respectively, and cloned into pTLR-SV40 vector along with either eGFP or Englong and Engshort coding sequence amplified using the following primers: eGFPPmeIfwd, 5′-GTTTAAACGCCACCATGGTGAGCA-3′; eGFPNotIrev, 5′-GCGGCCGCTTTACTTGTACA-3′; dreEngPmeIfwd, 5′-GTTTAAACTACAGCCGACATGAAGAGCA-3′; dreEngNotIrev, 5′-GCGGCCGCTCCTGTTGGATCAGGACGG-3′. Sequence-verified vectors were prepared and injected as a mix with transposase mRNA into one-cell-stage AB embryos as described in Kawakami et al. (2004). *fli1a:eGFP* transgenics were used to verify the EC-specific promotor activity of the *fli1a* promoter/enhancer throughout development to adulthood. Founder candidates were identified by PCR (dreEngSV40fwd, 5′-GAAGAGGAACGTGCAGGTCA-3′; SV40rev, 5′-GTTTCAGGTTCAGGGGGAGG-3′) and qPCR (dreEngqPCRgenofwd, 5′-TAACCCCGTCCTGATCCAAC-3′; dreEngqPCRgenorev, 5′-TGGCGTGAGGTTTGAGGTTT-3′; SV40rev, 5′-GTTTCAGGTTCAGGGGGAGG-3′) to detect endogenous and transgenic *endoglin*, respectively, using crude genomic DNA prepared from sperm as template. Transgene^+^ F1 fish carrying a single transgene copy were selected and crossed with *eng^+/−^* to produce *eng^+/−^*, *Tg(fli1a:engLong)* or *Tg(fli1a:engshort)* fish.

### Survival

Dead fish were collected and stored until final genotyping to confirm *eng^−/^*^−^ status and discriminate wild-type from heterozygous fish among the sibling group. Kaplan–Meier survival plots were generated and log-rank (Mantel–Cox) statistical analysis was performed using GraphPad Prism software.

### Imaging of blood vessel perfusion

At 72 hpf, GFP^+^ and dsRed^+^ siblings and *eng^−/−^* from crosses between *eng^+/−^, Tg(kdrl:GFP)* and *eng^+/−^, Tg(gata1:dsRed)* fish were anesthetized with ethyl 3-aminobenzoate methanesulfonate (MS222; 160 µg/ml; Sigma-Aldrich), mounted in 0.7% low-melt agarose and imaged on a Zeiss AXIO Zoom.V16 mounted with Zeiss AxioCam MRm with a Zeiss HXP 200C illuminator set on minimal power using fixed exposure parameters (GFP, 1 s; CY3, 1.5 s) to obtain red blood cell traces revealing perfusion extent. Similar postacquisition treatments were applied to images.

### Tissue clearing and deep imaging of whole zebrafish

To gain access to *in situ* dimensions of sibling and *eng^−/−^* heart ventricle at 10, 12 and 15 dpf, clutches from crosses between *eng^+/−^, Tg(cmlc2:GFP)* and *eng^+/^*^−^ fish were screened at 24 dpf to sort out GFP^+^ individuals and at 72 dpf to discriminate siblings from *eng^−/−^* fish. Fish were raised as described earlier, collected at indicated times by adding excess MS222 (320 μg/ml) and fixed overnight in 4% PFA. Whole zebrafish were depigmented and labeled according to a previously published protocol (Frétaud et al., 2021) using rabbit anti-mCherry (600-401-P16, Rockland) and chicken anti-GFP (A10262, Thermo Fisher Scientific) prior to Alexa Fluor 594 goat anti-rabbit (A11012, Thermo Fisher Scientific) and Alexa Fluor 488 goat anti-chicken (A11039, Thermo Fisher Scientific). Before imaging, larvae were cleared by incubation in RIMS (Yang et al., 2014) overnight at room temperature. Larvae were mounted under #1 coverslips in RIMS supplemented with 0.8% low-gelling agarose. Images were acquired with a Leica SP8 confocal microscope using a HCX IRAPO L 25×/0.95 NA water immersion objective (11506340, Leica Microsystems). Ventricle volume (*V*) is estimated by the simplified ellipsoid volume calculation formula *V*=0.523(width in mm)^2^(length in mm) (Hoage et al., 2012).

### Confocal imaging

GFP/Tomato^+^ embryos from crosses between *eng^+/−^, Tg(kdrl:GFP)* and *eng^+/^*^−^*, Tg(flt1:Tomato)* zebrafish were screened at 72 hpf to discriminate siblings from *eng^−/−^* zebrafish. Fish were raised as described earlier, collected at indicated times by adding excess MS222 (320 μg/ml) and fixed overnight in 4% PFA. Fixed zebrafish were mounted in 0.7% low-melt agarose in a Fluorodish glass-bottom culture dish (WPI) and imaged on a Zeiss LSM510 confocal microscope. Dissected organs from 29 dpf *eng^−/−^* fish or siblings were imaged on a Zeiss LSM880 confocal microscope. Projections of *z*-stacks were performed using Fiji software. Similar postacquisition treatments were applied to images. Blood vessel measurements were performed using Fiji software.

### Flow cytometry

GFP^+^ siblings and *eng^−/−^* fish from crosses between *eng^+/−^, Tg(cmlc2:EGFP)* and *eng^+/−^* fish were sorted at 3 dpf and raised as described above. At 5, 10, 15, 20 and 25 dpf, fish were collected as pools of 26, 18, nine, seven and seven fish, respectively, and dissociated in 0.25% Trypsin-EDTA (Gibco, Thermo Fisher Scientific) and 8 mg/ml collagenase from *Clostridum histolyticum* (Sigma-Aldrich) according to a previously published procedure (Bresciani et al., 2018). Dissociated cells were fixed overnight in 4% PFA at 4°C, then rinsed thrice in PBS and stored at 4°C in PBS, 0.05% sodium azide until analysis. Samples (250,000 cells) were analyzed on a FACSCanto cytofluorimeter (Becton Dickinson) at indicated time points to evaluate cardiomyocyte (GFP^+^) representation in whole-fish cell suspensions. FACS profiles were validated using cardiomyocyte-enriched cell suspensions prepared from GFP^+^ isolated *Tg(cmlc2:EGFP)* hearts.

### Phz treatment

Wild-type zebrafish (30 fish per group) were bathed every other day from 14 dpf with 1.25, 2.5 and 5 µg/ml phz in fish water prepared from a freshly made 5 mg/ml stock solution of phz (Sigma-Aldrich). Non-treated fish served as control. Bathing volume was gradually increased to accommodate fish growth (100 ml to 300 ml). Fish were treated for 30 min, then allowed to recover in fresh fish water for ∼1 h, and finally replaced into housing tanks and fed. Fish were subjected to this regimen up to 75 dpf before treatment was definitively stopped to let fish recover and regain sex-specific hallmarks to allow for accurate sex ratio determination. To assess hypoxia and cardiac stress in fish under phz treatment, five samples constituted of five fish for each condition were collected at 29 dpf (treatment-free day) and euthanized with excess MS222 (320 μg/ml) for total RNA extraction performed as described above. Phz treatment of *eng^−/−^* and siblings was performed as for wild-type (see above) but phz concentrations were downscaled to 0.625, 1.25 and 2.5 µg/ml. Non-treated *eng^−/−^* fish and siblings were used as control. At 27 dpf (treatment-free day), each condition was divided into six samples (four fish/sample) and used for total RNA extraction. For phz effect on *eng^−/−^* survival, *eng^−/−^* fish and siblings were treated as described above using 0.625 and 1.25 mg/ml phz up to 2 months, and were left to recover for 15 days to allow for sex determination.

### Histology

Fish were euthanized with excess MS222 (320 μg/ml) and fixed overnight in 4% PFA. Samples were decalcified using 0.35 M EDTA. Paraffin embedding was performed by the Réseau d'Histologie Expérimentale de Montpellier (RHEM) Histology Facility. Sagittal sections (7 µm) were dewaxed and stained by Hematoxylin-Eosin (H&E). Fiji software was used for heart measurements. Whole-mount *in situ* hybridization samples were postfixed overnight in 4% PFA before paraffin embedding. Sagittal and transversal sections (7 µm) were dewaxed and counterstained with Nuclear Fast Red.

### Hemoglobin staining, blood smears and hematocrit measurement

Hemoglobin content in 3, 5 10 and 15 dpf *eng^−/−^* fish and siblings was assessed using o-dianisidine reagent according to a previously published procedure (Leet et al., 2014). Briefly, embryos and larvae were stained with 0.6 mg/ml o-dianisidine (Alfa Aesar) in 10 mM sodium acetate pH 4.5, 0.65% H_2_O_2_ and 40% (v/v) ethanol for 30 min at room temperature. Fish were then washed with PBS and fixed overnight in 4% PFA. The next day, fish were washed with PBS and soaked for 30 min in 0.8% KOH, 0.9% H_2_O_2_ and 0.1% Tween 20 to remove pigments. After washes with PBS, fish were scored for hemoglobin staining, and pictures of representative fish were taken after mounting in 0.7% low-melt agarose. For blood smears, fish were anaesthetized using MS222 (160 µg/ml), and blood samples obtained from cardiac puncture were smeared on microscope slides. Wright-Giemsa staining was performed following the manufacturer's instructions (Sigma-Aldrich). For hematocrit measurement, wild-type and *eng^−/−^* adult fish were anaesthetized as described above, and blood samples were obtained by cardiac puncture using 18 µl heparinized capillary tubes (Hirschmann). Samples were processed using Hematocrit 24 centrifuge (Hettich), and hematocrit was assessed.

### Statistics

Kaplan–Meier plots for survival were analyzed using log-rank (Mantel–Cox) test. With the exception of frequency analyses, all other values in this study were expressed as mean±s.e.m., and statistical significance was analyzed by Mann–Whitney test, unpaired one-tailed Student's *t*-test, Kruskal–Wallis test, Brown–Forsythe test and Welch’s test or one-way ANOVA after assessing sample normality and similar variance. For multiple comparisons, Benjamini, Krieger and Yekutieli posthoc test was used. Sample/group sizes were defined based on preliminary experiments. Investigators were usually aware of group allocation during sample collection and analysis. No animals were excluded from analyses. *P*≤0.05 was considered significant.

## Supplementary Material

10.1242/dmm.049488_sup1 Supplementary information
