# Supplementary material for: Deficiency in hereditary hemorrhagic telangiectasia-associated Endoglin elicits hypoxia-driven heart failure in zebrafish
Source: Dis Model Mech. 2023 Jun 2;16(5):dmm049488. doi: 10.1242/dmm.049488 (PMC10245139; doi:10.1242/dmm.049488)
Supplement: Supplementary information [file dmm-16-049488-s1.pdf]

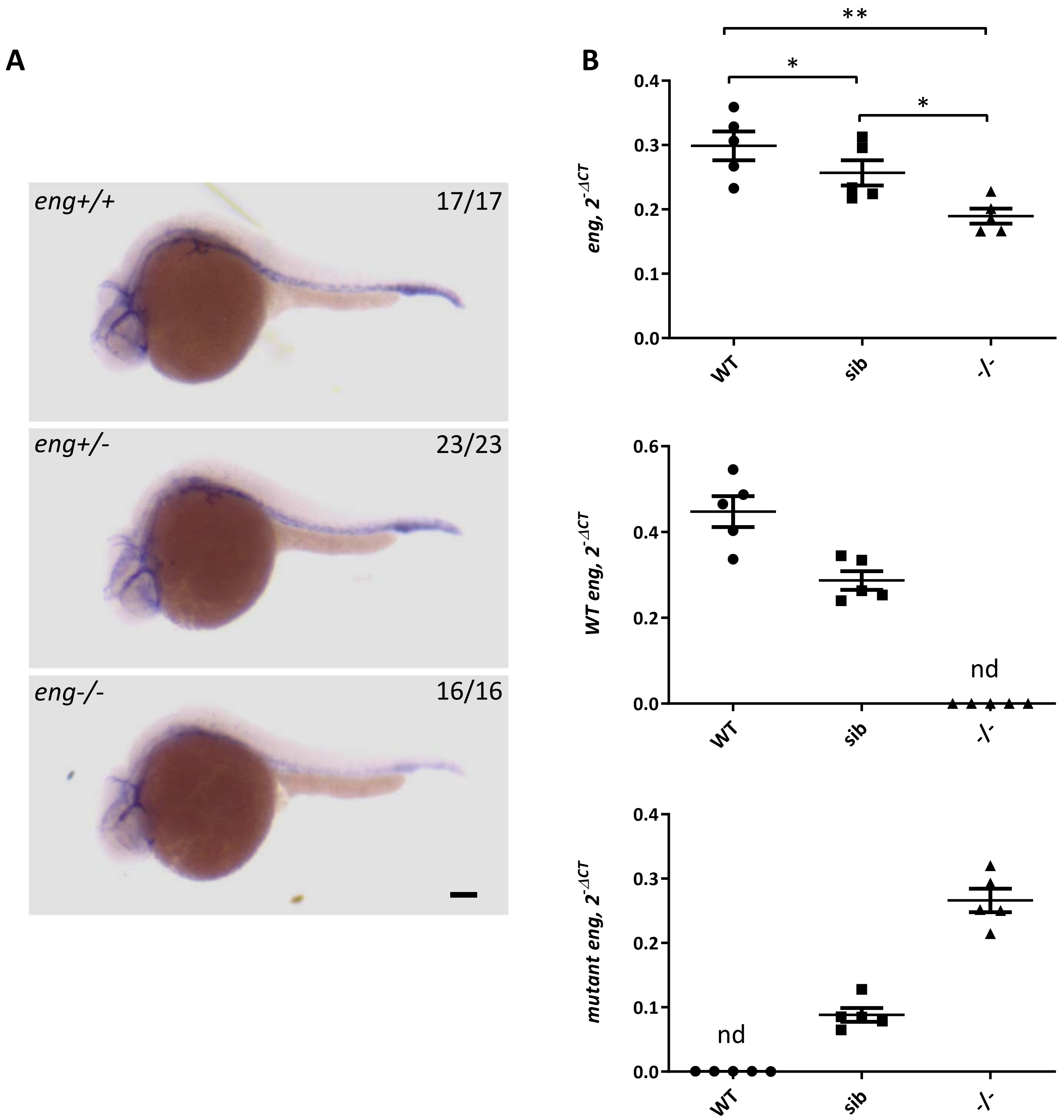

Fig. S1. Decreased amounts of endoglin transcripts in *eng*$^{-/-}$. (A) Representative pictures of whole mount *in situ* hybridization using *eng* antisense riboprobe on 26hpf embryos from *eng*$^{+/-}$ incrosses. Upper right markings indicate the number of fish displaying similar features over the number of fish analyzed. Bar, 100µm. (B) Quantitative RT-PCR analysis of all-endoglin (upper panel), wild-type endoglin (middle panel) and mutant endoglin (lower panel) mRNA expression in 72hpf wild-type (WT), siblings (sib) and *eng*$^{-/-}$ (-/-) embryos. Target gene expression is represented as $2^{-\Delta CT}$ using *rpl13a* as reference. Samples (5 for each genotype) are pools of 17 to 23 embryos. nd, not detected. Statistical analysis: one-way ANOVA and two-stage linear set-up procedure of Benjamini, Krieger and Yekutieli post-hoc test for multiple comparisons after assessment of normal distribution and equal SD. FDR adjusted *P* values; WT vs sib *P*=0.047, WT vs -/- *P*=0.0013 and sib vs -/- *P*=0.0125.

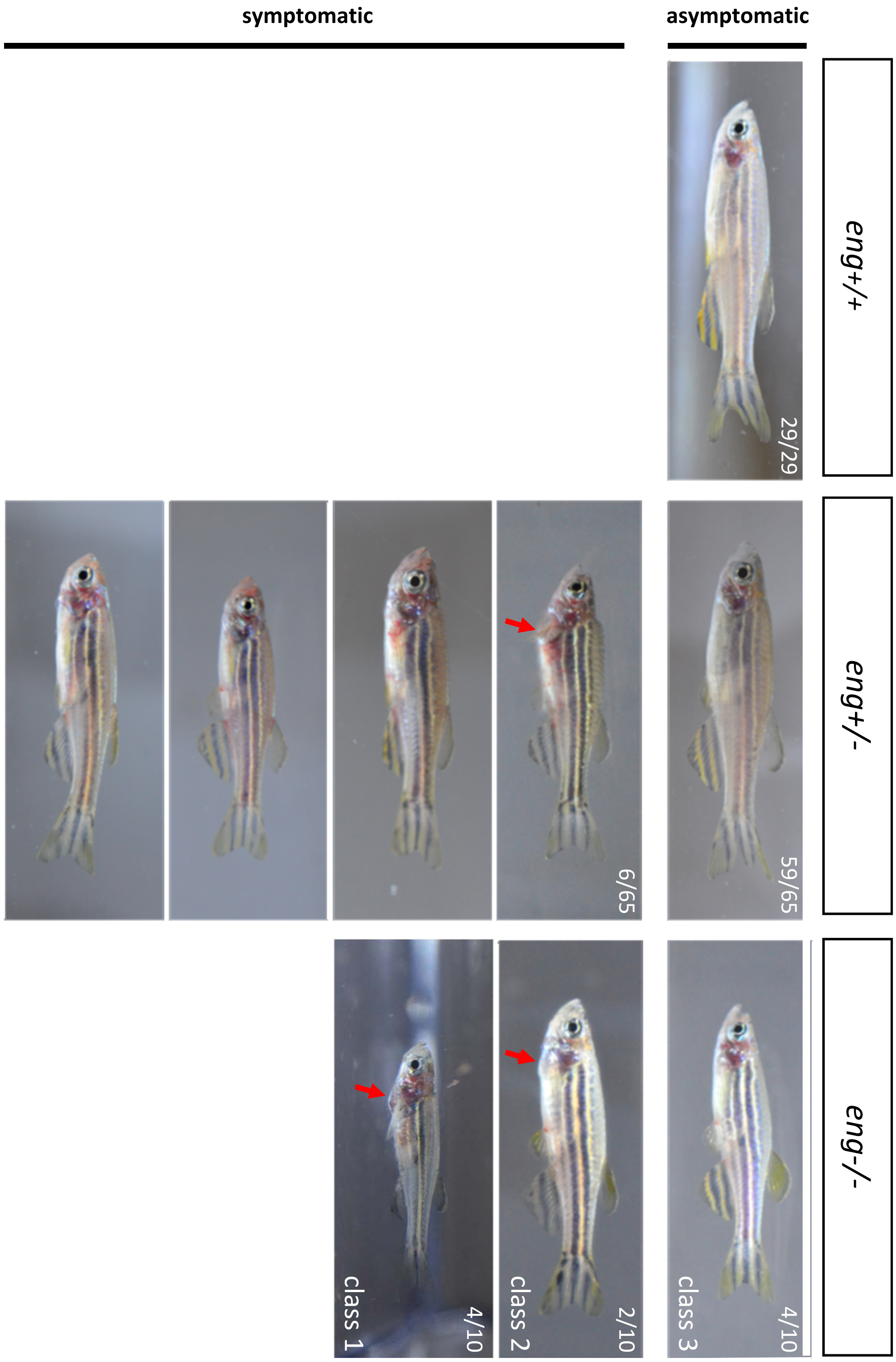

Fig. S2. Overall appearance of 4-month-old *eng*$^{+/+}$, asymptomatic and symptomatic *eng*$^{+/-}$ and *eng*$^{-/-}$ fish. Note the overall reddish color of symptomatic fish and dilated blood vessels at the root of caudal, pectoral and anal fins. Severity classes of *eng*$^{-/-}$ assigned as follow 1) symptomatic juvenile size fish. 2) symptomatic adult size fish and 3) asymptomatic fish. Arrow points to enlarged cardiac area.

Upper right markings indicate the number of fish displaying similar features over the number of fish analyzed. Pictures were taken from alive fish directly in their housing tanks therefore fish size is not representative.

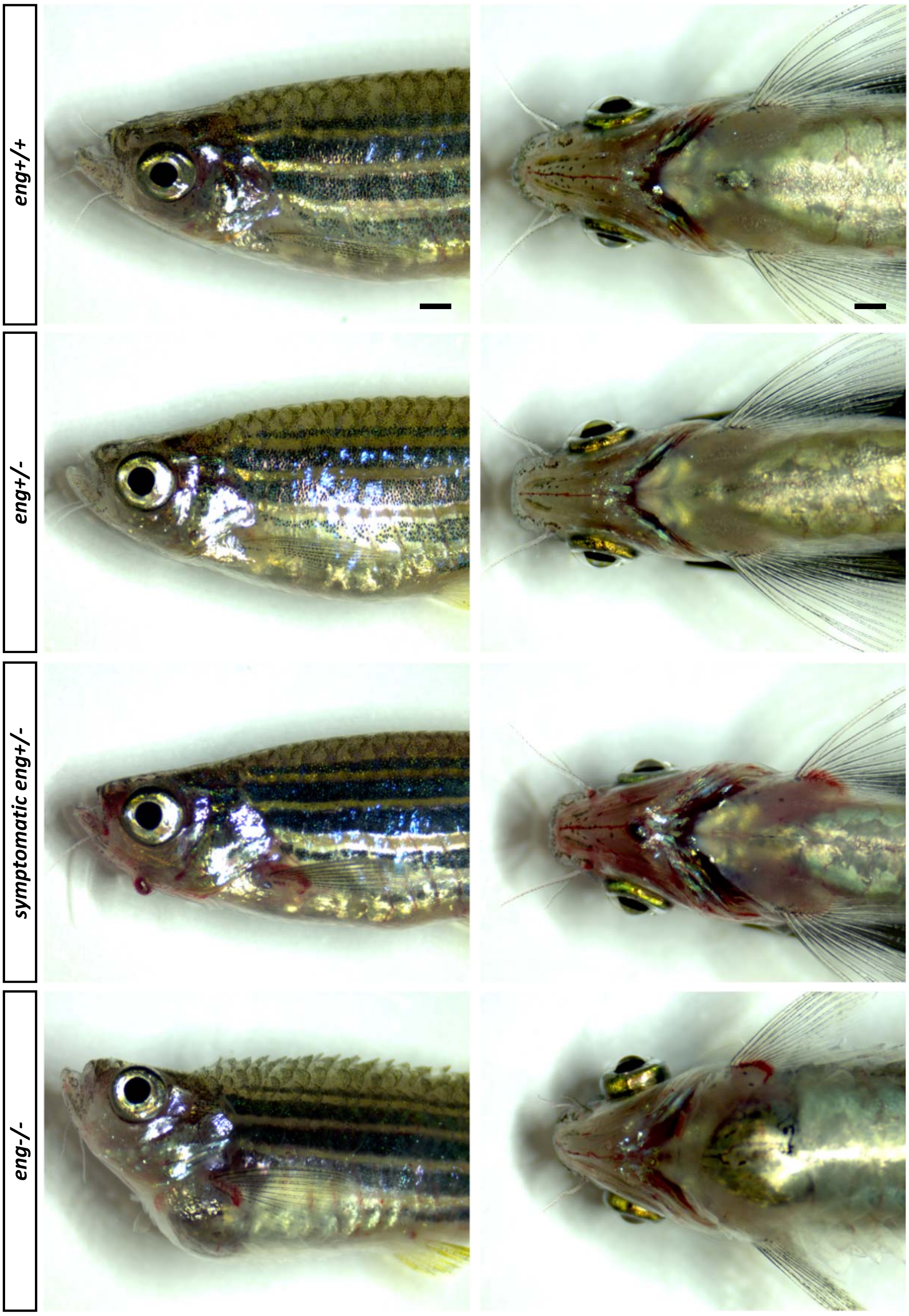

Fig. S3. Phenotypic details of symptomatic *eng*$^{+/-}$. Representative lateral (left panels) and ventral (right panels) pictures of adult (6 months) *eng*$^{+/+}$, asymptomatic and symptomatic *eng*$^{+/-}$ and *eng*$^{-/-}$ (females) showing the presence of dilated blood vessels reminiscent of telangiectasias. Note the cardiomegaly in *eng*$^{-/-}$ and characteristic hydropsy symptoms such as bulging eyes and pinecone-like scales, sign of multiple organ failure only observed secondary to heart failure. Bar, 1 mm.

Disease Models & Mechanisms • Supplementary information

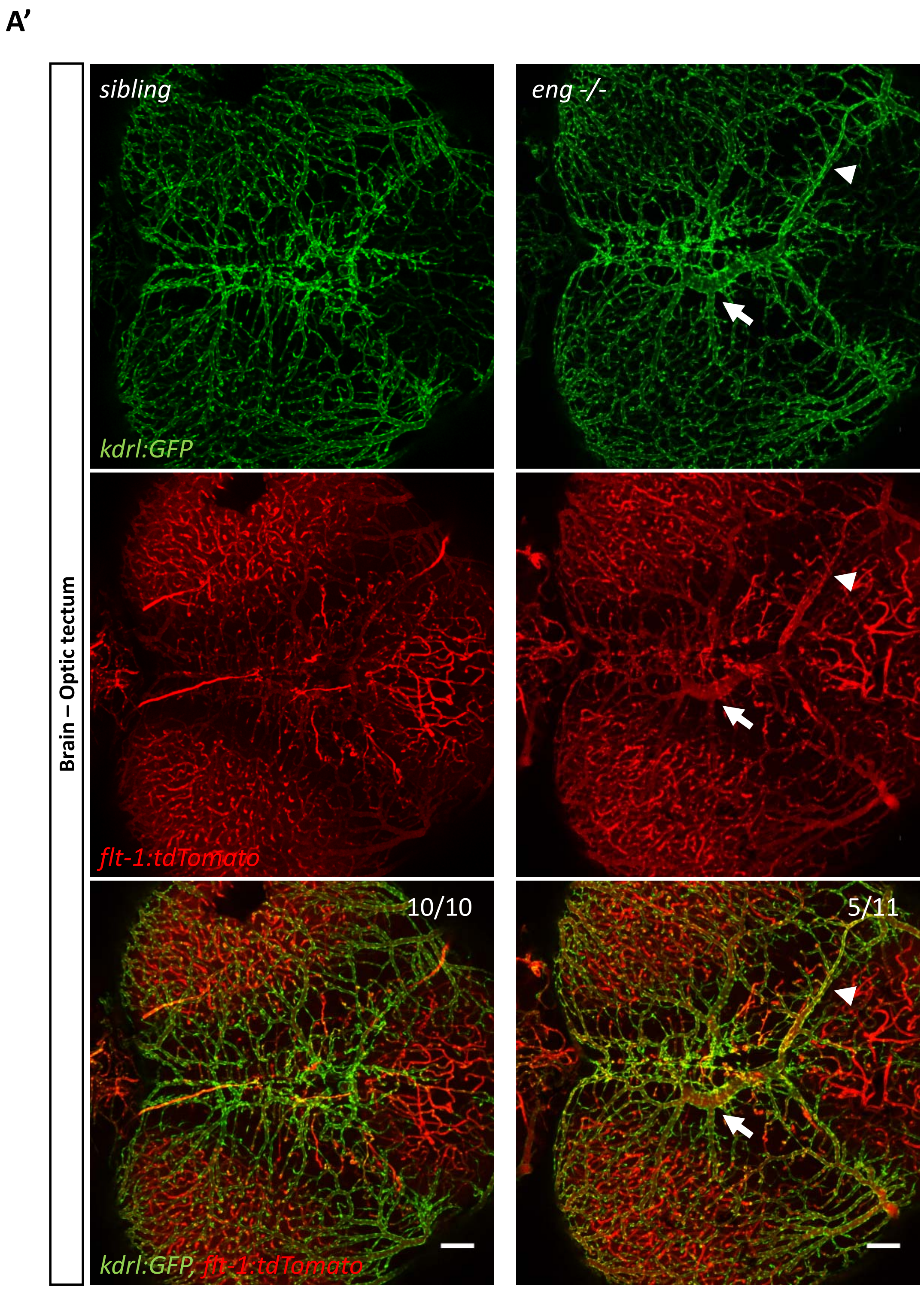

Disease Models & Mechanisms • Supplementary information

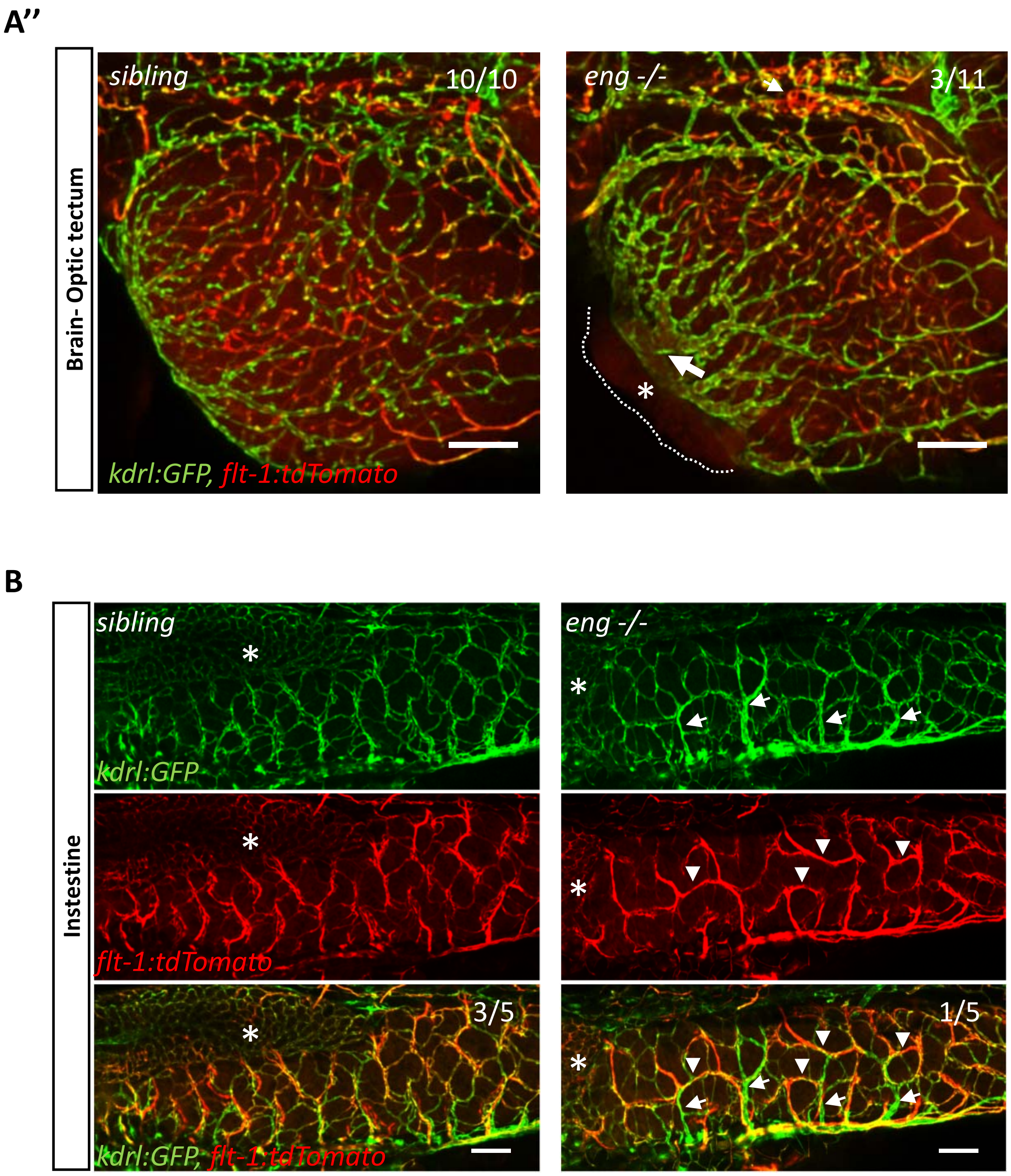

Disease Models & Mechanisms • Supplementary information

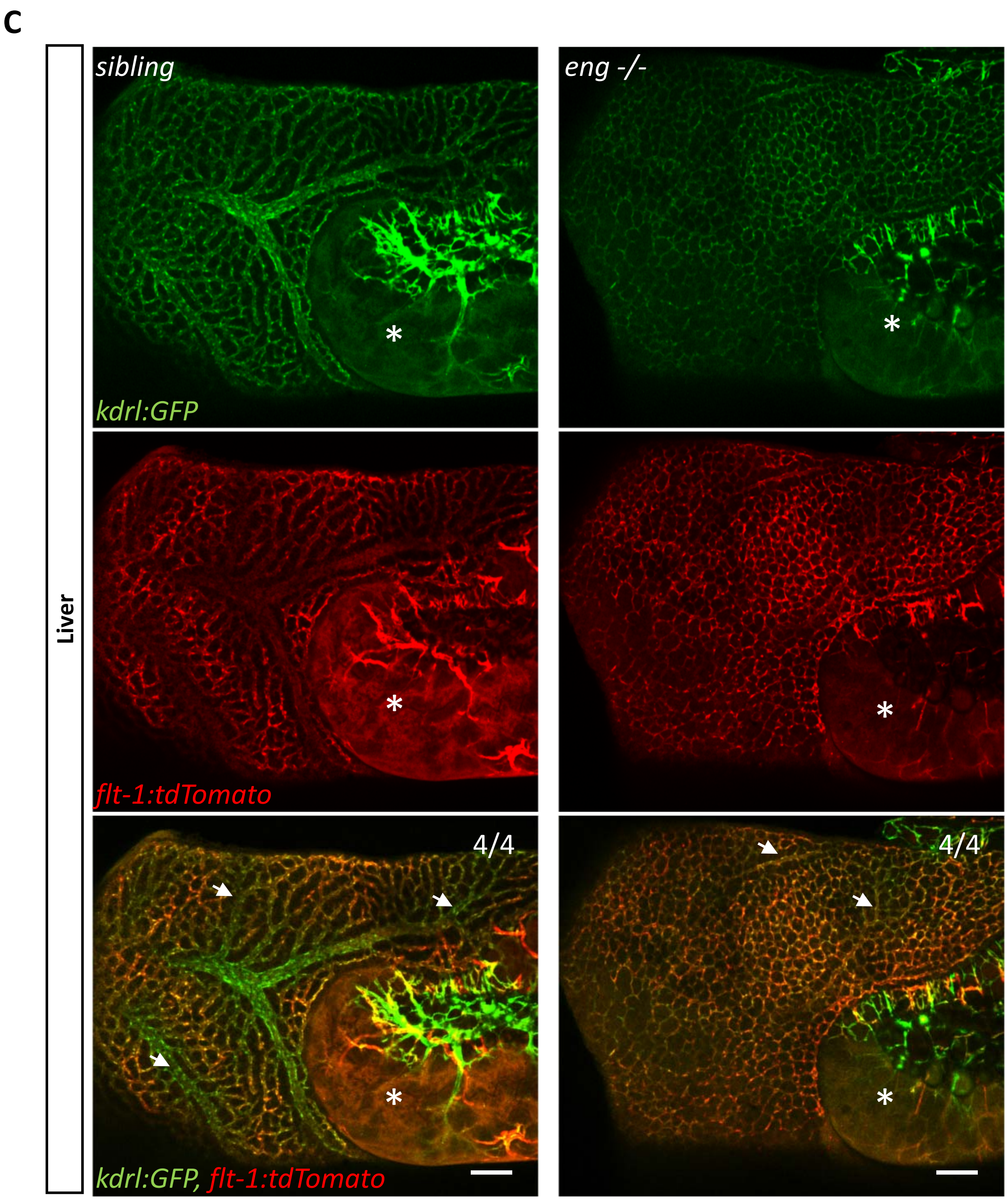

Fig. S4. Vascular defects in *eng*$^{-/-}$ fish brain, intestine and liver. Confocal imaging of dissected organs from 29dpf sibling and *eng*$^{-/-}$ fish in *Tg(kdrl:GFP)*, *Tg(flt1:tdtomato)* background. (A') Brain vascular architecture in optic tectum area. Representative pictures of vascular defects found in *eng*$^{-/-}$ fish i.e. enlarged and contorted vein (arrow) and artery directly connected to veins (arrowhead). (A'') Large venous malformation (arrow) associated with hemorrhage (asterisk) evidenced by erythrocyte autofluorescence. (B) Intestine vascular architecture in caudal intestine area. Dilated veins (arrows) can be observed in *eng*$^{-/-}$. Also note the tendency of arteries to connect neighbors through lateral branches and the locally low capillary density. Liver (asterisk) . (C) Liver vascular architecture. Representative pictures of the unusual honeycomb-like organization of blood vessels and the poor artery-vein structural and molecular hierarchy in *eng*$^{-/-}$ fish as opposed to the regular tree-like organization and clear artery/vein distinction by *flt-1* reporter expression levels. Veins (arrows). Intestine (asterisk). Upper right markings indicate the number of fish displaying similar features over the number of fish analyzed. Bar, 100µm.

Disease Models & Mechanisms • Supplementary information

A

B

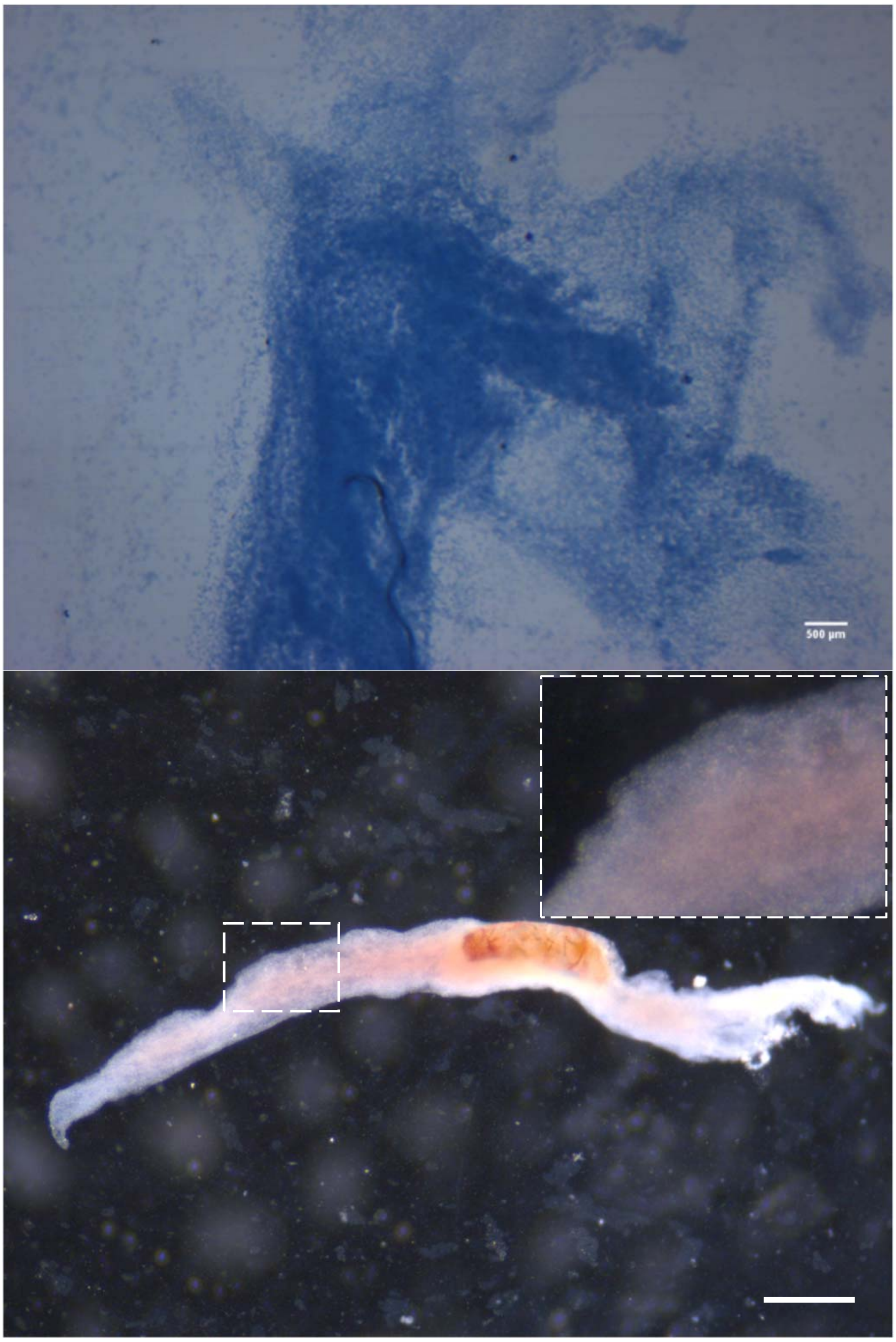

Fig. S5. Intestinal hemorrhages in adult *eng*$^{-/-}$ fish. (A) Wright-Giemsa stained smear of a clot collected after intestinal hemorrhage in a 5 month old *eng*$^{-/-}$ fish. The smear (only part of it) is shown as an indication of blood loss extent. Bar, 100µm. (B) Fresh clot from intestinal hemorrhage in 2-month-old *eng*$^{-/-}$ fish. Bar, 1mm. Inset, close-up picture. Note the size of the sample indicative of massive blood loss. Clot rapidly turns pale since erythrocytes tends to release their content once exposed to tank water. Fish died 4 days after hemorrhagic episode.

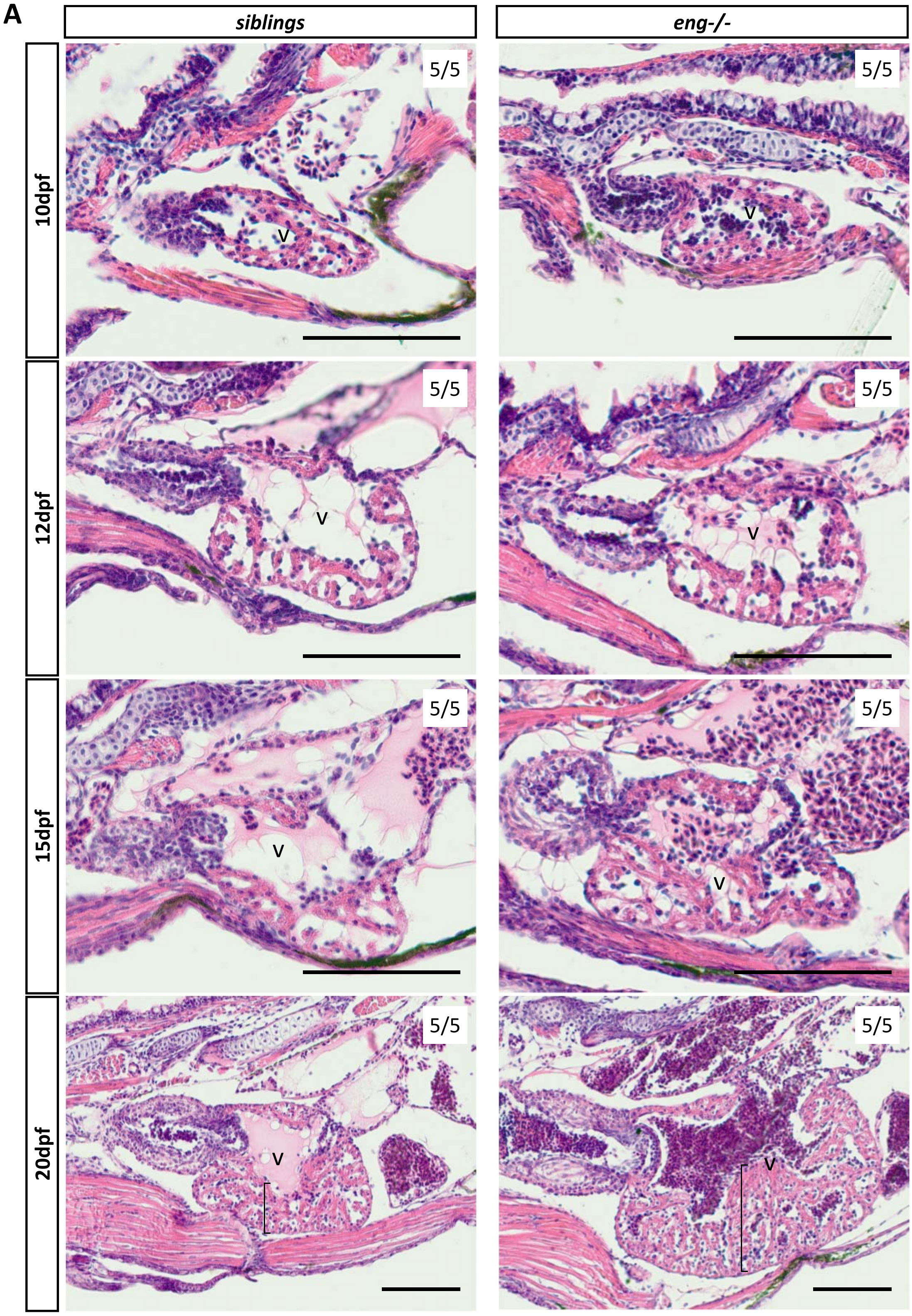

Fig. S6. Kinetic analysis of *eng*$^{-/-}$ heart structural modifications. (A) Representative pictures of H&E stained histological sections from 10, 12, 15 and 20dpf siblings and *eng*$^{-/-}$. Numbers in the upper right corner of pictures indicate the number of fish with similar features over the total number of fish analyzed. Brackets indicates the area where myocardium thickness was measured. Bar, 100µm. (v, ventricle). Note ventricle enlargement and erythrocyte filled cardiac chambers by 15dpf. (B) Measurements of siblings and *eng*$^{-/-}$ ventricle at 20dpf. Upper panel, ventricle wall thickness in µm, *P*= 0.0037. Middle panel, ventricle size evaluated from ventricle area in µm^2, *P*= 0.0009. Lower panel, thickness/ heart size ratio, *P*= 0.074. Number of individual analyzed per group n= 5. Statistical analysis, one tailed unpaired *t*-test after assessment of normality and equal SD.

Disease Models & Mechanisms • Supplementary information

B

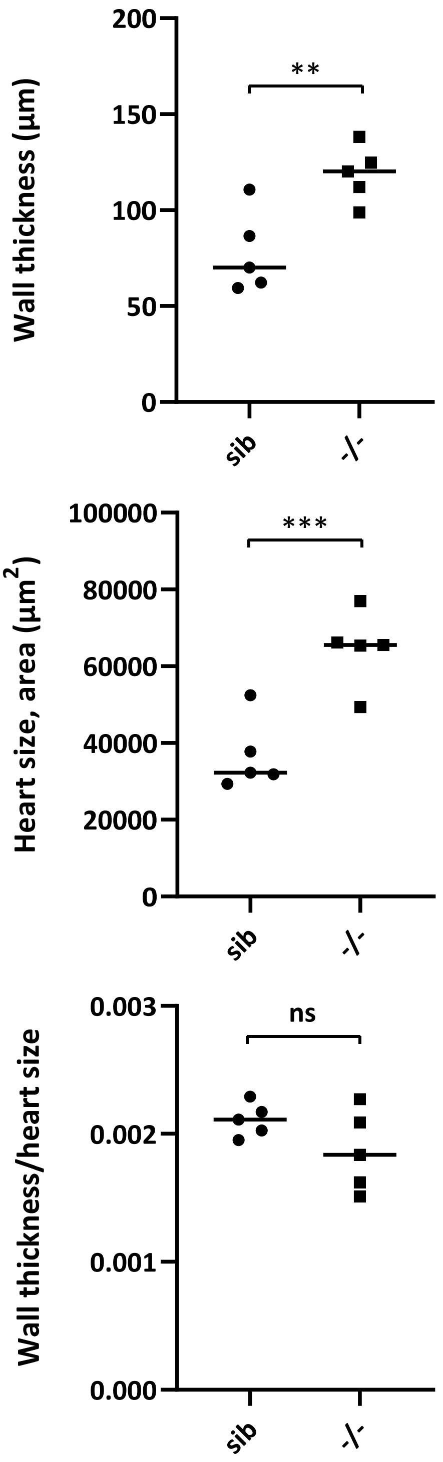

Disease Models & Mechanisms • Supplementary information

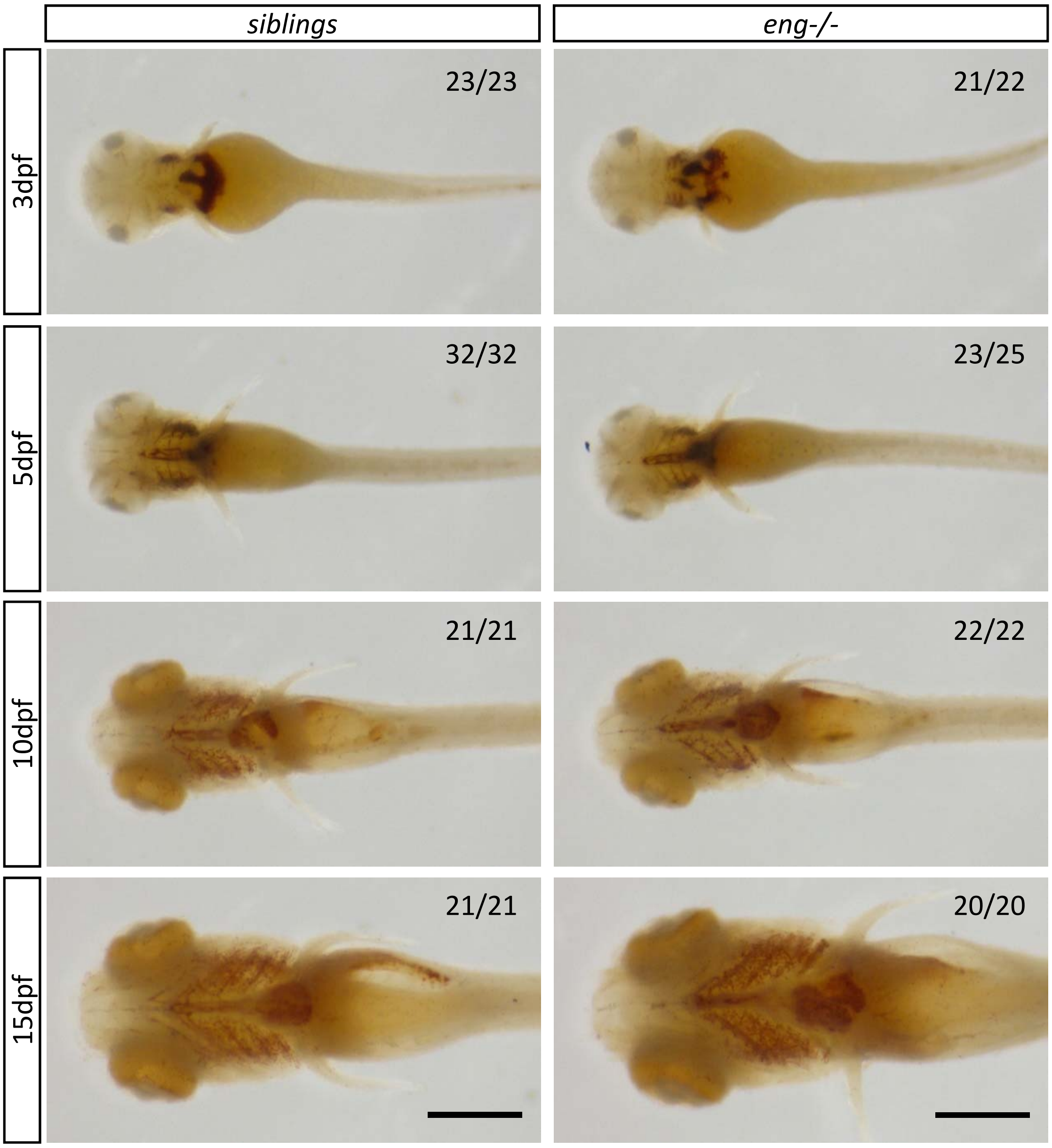

Fig. S7. Hemoglobin assessment does not reveal early anemia in *eng*$^{-/-}$ fish. Representative whole-mount o-dianisidine staining of 3, 5, 10 and 15dpf *eng*$^{-/-}$ and siblings. Note the absence of decreased staining in *eng*$^{-/-}$ compared with siblings. Conversely, note the higher staining in 15dpf mutant fish. Upper right markings indicate the number of fish displaying similar features over the number of fish analyzed. Bar, 500 µm.

Disease Models & Mechanisms • Supplementary information

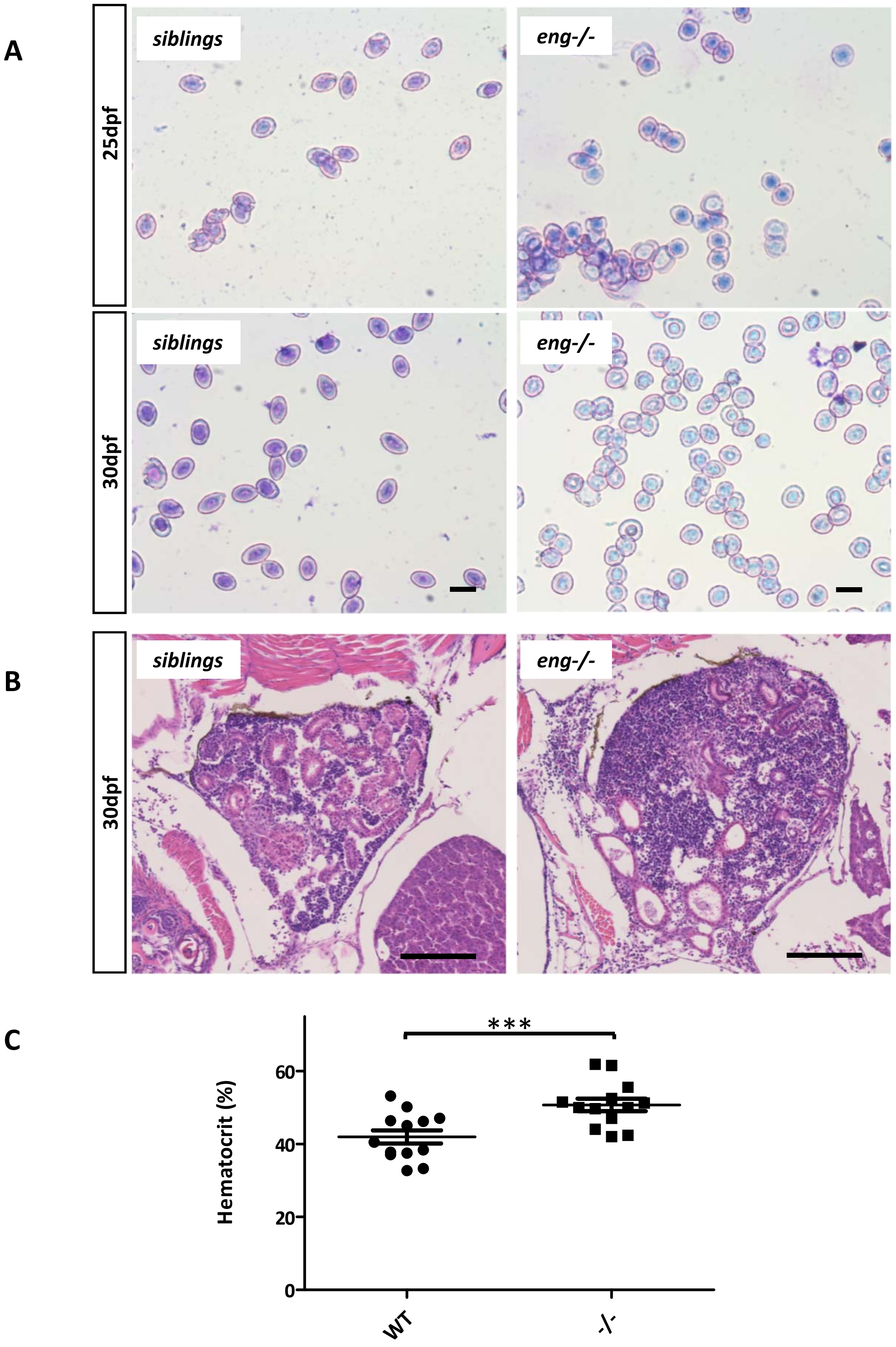

Fig. S8. Hematological features of *eng*$^{-/-}$ fish. (A) Representative pictures of Wright-Giemsa stained blood smears from siblings versus *eng*$^{-/-}$ fish at 25 and 30dpf. Note the marked difference of erythrocyte shape and staining between siblings and *eng*$^{-/-}$. Bar, 20 µm. (B) Representative pictures of H&E stained histological sections of kidney from siblings versus *eng*$^{-/-}$ fish at 30dpf. Note kidney hypercellularity indicative of reactive erythropoiesis. Bar, 100 µm. (C) Adult (6 month or older) *eng*$^{-/-}$ exhibit increased hematocrit compared to wild-type fish. Mean hematocrit of wild-type (n=13) 41.96 ± 1.798 % vs *eng*$^{-/-}$ (n=13) 50.75 ± 1.746 %, *P* =0.0009. Statistical analysis: one-tailed unpaired *t*-test, after assessment of normality and equal SD

Disease Models & Mechanisms • Supplementary information

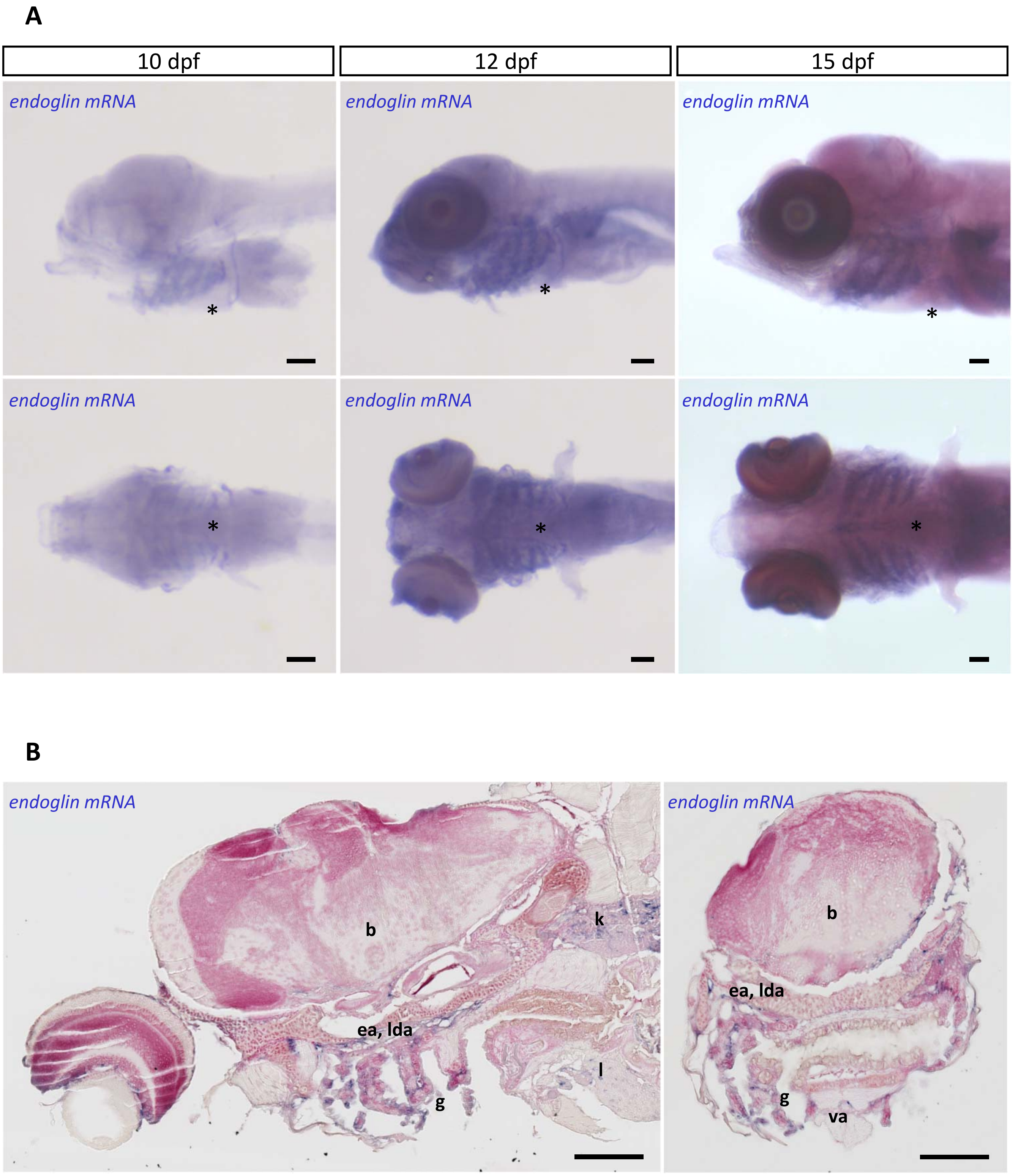

Fig. S9. Endoglin expression in developing gills. (A) Whole-mount *in situ* hybridization using *endoglin* antisense riboprobe on 10, 12 and 15dpf wild-type zebrafish larvae. Upper panels, lateral views; lower panels, ventral views. Bar, 100 µm. Asterisk indicates heart location. (B) Representative histological sections of WISH using *endoglin* antisense probe in 15dpf zebrafish larvae showing *endoglin* expression in developing gills. Sagittal section (left) transversal section (right) (b,brain; ea-lda, epibranchial arteries-lateral dorsal aorta; g, gills k, kidney; l, liver, va, ventral aorta). Bar, 100 µm.

Disease Models & Mechanisms • Supplementary information

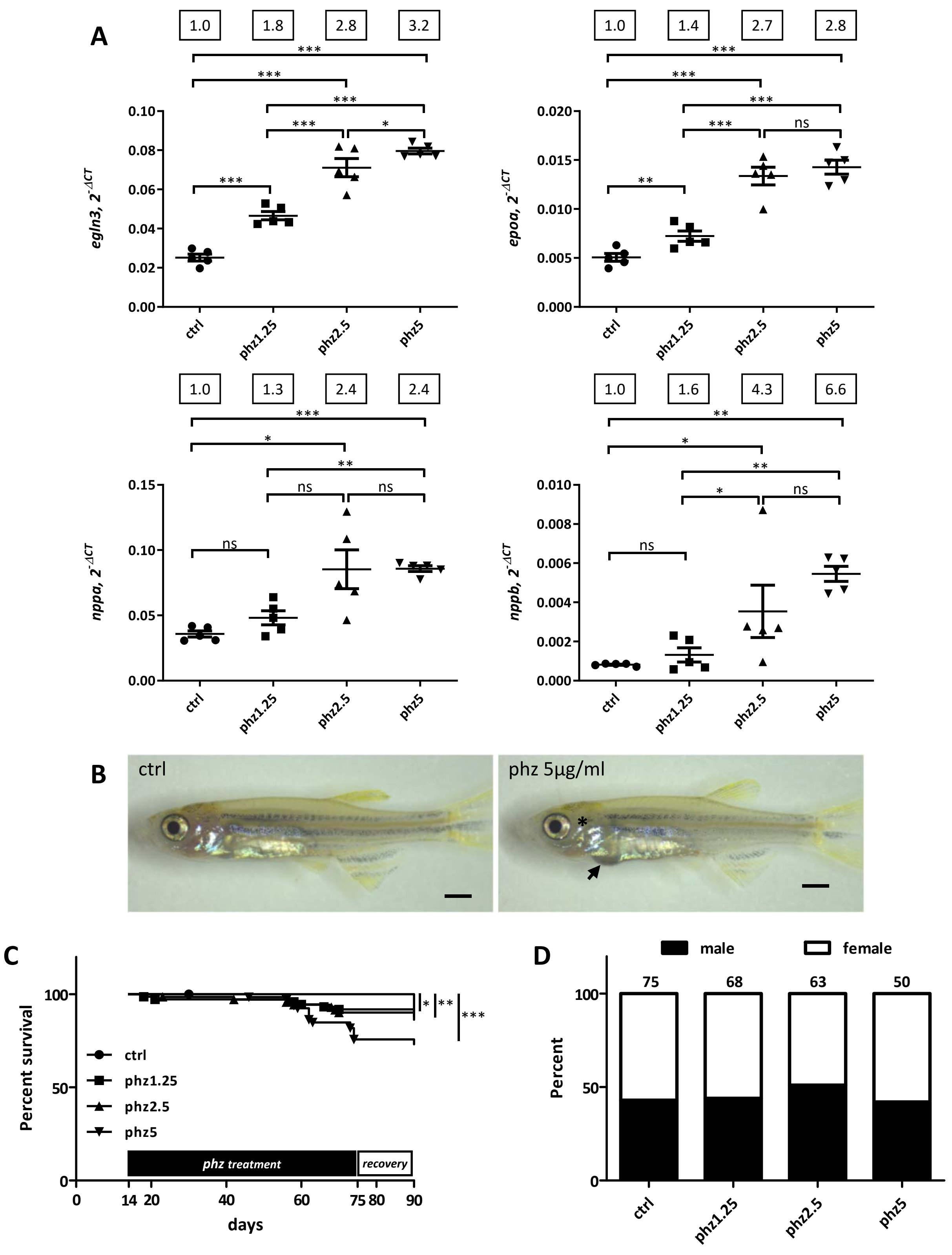

Disease Models & Mechanisms • Supplementary information

Fig. S10. Phenylhydrazine-induced hypoxia fails to mimic *eng*$^{-/-}$ heart failure condition. (A) RT-qPCR analysis of *egln3*, *epoa* (hypoxia responsive gene) and *nppa* and *nppb* (cardiac stress responsive gene) expression in one-month-old wild-type fish non-treated (ctrl) or treated with 1.25, 2.5 and 5 µg/ml phenylhydrazine (phz). Target gene expression is represented as $2^{-\Delta CT}$ using *rpl13a* as reference. Mean fold change is indicated above graphs. Samples (n=5) are pools of 5 fish. Data are presented as individual values and mean ± SEM. Statistical analysis: one-way ANOVA, Brown-Forsythe and Welch ANOVA (*nnpa*) or Kruskal-Wallis test (*nppb*) and two-stage linear set-up procedure of Benjamini, Krieger and Yekutieli post-hoc test for multiple comparisons after assessment of normal distribution and equal SD. FDR adjusted *P* values; *egln3*, ctrl vs phz1.25 *P*<0.0001, ctrl vs phz2.5 *P*<0.0001, ctrl vs phz5 *P*<0.0001, phz1.25 vs phz2.5 *P*<0.0001, phz1.25 vs phz5 *P*<0.0001, phz2.5 vs phz5 *P*=0.0498. *epoa*, ctrl vs phz1.25 *P*=0.0072, ctrl vs phz2.5 *P*<0.0001 , ctrl vs phz5 *P*<0.0001, phz1.25 vs phz2.5 *P*<0.0001, phz1.25 vs phz5 *P*<0.0001, phz2.5 vs phz5 *P*=0.3524. *nppa*, ctrl vs phz1.25 *P*=0.0702, ctrl vs phz2.5 *P*=0.0391, ctrl vs phz5 *P*<0.0001, phz1.25 vs phz2.5 *P*=0.0682, phz1.25 vs phz5 *P*=0.0021, phz2.5 vs phz5 *P*=0.6820. *nppb*, ctrl vs phz1.25 *P*=0.3718, ctrl vs phz2.5 *P*=0.0226, ctrl vs phz5 *P*=0.042, phz1.25 vs phz2.5 *P*=0.0428, phz1.25 vs phz5 *P*=0.0073, phz2.5 vs phz5 *P*=0.2290. (B) Representative pictures of one-month-old wild-type fish non-treated (ctrl) or treated with 5 µg/ml phenylhydrazine (phz). Note the overt paleness of gill area (asterisk) and slightly enlarged heart region (arrow). Bar, 1 mm. (C) Analysis of phenylhydrazine treatment effect over fish survival. Kaplan-Meyer representation of the survival of non-treated (ctrl) and 1.25, 2.5 and 5 µg/ml phz treated wild-type fish. Note the dose dependent effect of phz concentration over survival but absence of deleterious effect at early juvenile .0114, ctrl vs phz

Disease Models & Mechanisms • Supplementary information

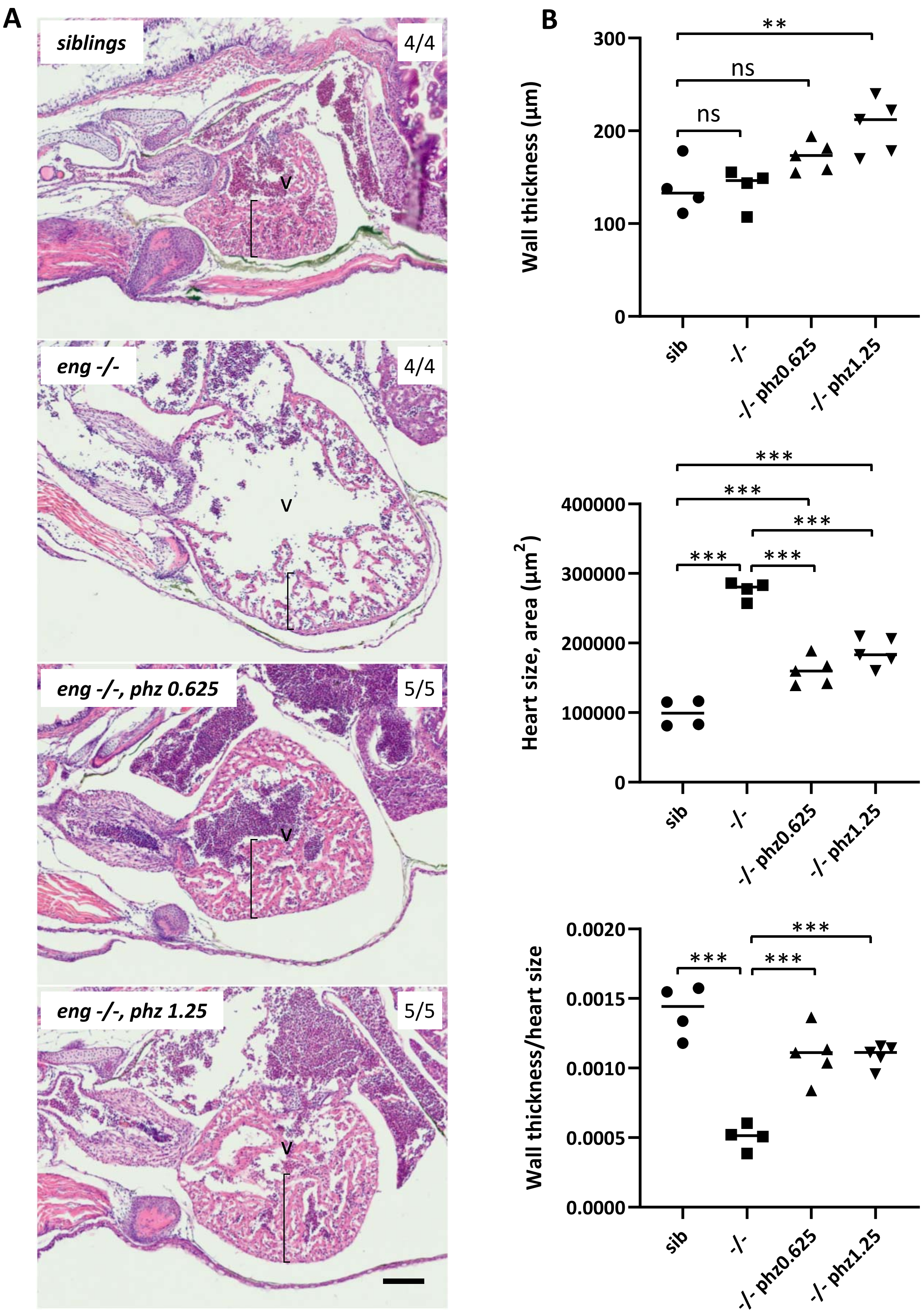
A
siblings
4/4
v
eng -/-
4/4
v
eng -/-, phz 0.625
5/5
v
eng -/-, phz 1.25
5/5
v
B
Wall thickness (μm)
300
200
100
0
**
ns
ns
sib
-/-
-/- phz0.625
-/- phz1.25
Heart size, area (μm2)
400000
300000
200000
100000
0
***
***
***
***
***
Wall thickness/heart size
0.0020
0.0015
0.0010
0.0005
0.0000
***
***
***

Fig. S11. Phenylhydrazine treatment of *eng*$^{-/-}$ fish limits cardiomegaly and preserves heart internal structure. (A) Representative pictures of H&E stained histological sections of heart area from 27dpf siblings, *eng*$^{-/-}$ and *eng*$^{-/-}$ treated with 0.625 and 1.25 µg/ml phenylhydrazine. Numbers in the upper right corner of pictures indicate the number of fish with similar features over the total number of fish analyzed. Brackets indicates the area where myocardium thickness was measured. Bar, 100 µm. (v, ventricle). (B) Measurements of ventricle from 27dpf siblings, *eng*$^{-/-}$ and phz treated *eng*$^{-/-}$ fish. Upper graph, ventricle wall thickness (in µm). Middle graph, heart size (ventricle area in µm^2). Lower graph, thickness/heart size ratio. Statistical analysis: one-way ANOVA and two-stage linear set-up procedure of Benjamini, Krieger and Yekutieli post-hoc test for multiple comparisons after assessment of sample normal distribution and equal SD. Wall thickness, FDR adjusted *P* values, sib vs -/- *P*=0.6993, sib vs -/- phz0.625 *P*=0.0507, sib vs -/- phz1.25 *P*=0.0028. Heart size, FDR adjusted *P* values, sib vs -/- *P*<0.0001, sib vs -/- phz0.625 *P*=0.0004, sib vs -/- phz1.25 *P*<0.0001, -/- vs -/- phz0.625 *P*<0.0001, -/- vs -/- phz0.625 *P*<0.0001. Wall thickness/heart size FDR adjusted *P* values, sib vs -/- *P*<0.0001, sib vs -/- phz0.625 *P*=0.0014, sib vs -/- phz1.25 *P*=0.0014

Disease Models & Mechanisms • Supplementary information

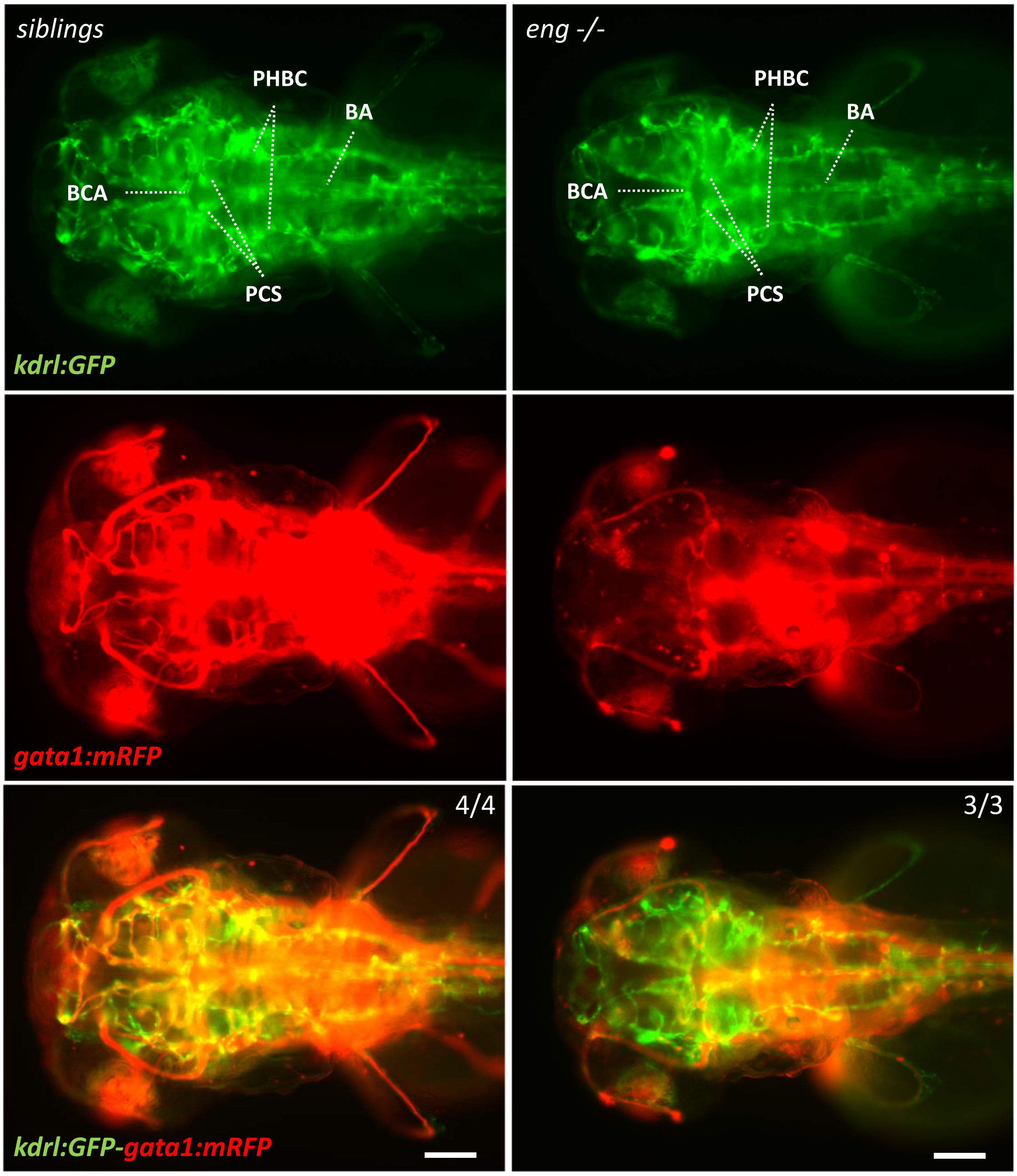

Fig. S12. Absence of *alk1* mutant-like vascular defects in *eng*$^{-/-}$ fish. Imaging of 72hpf sibling and *eng*$^{-/-}$ zebrafish brain blood vessel architecture and perfusion. Analysis performed in *Tg(kdrl:GFP)*, *Tg(gata1:mRFP)* background to highlight blood vessels and erythrocytes, respectively. Note the normal caliber blood vessels such as basal communicating artery (BCA), posterior connecting segments (PCS), basilar artery (BA) and primordial hindbrain Channel (PHBC) reported as enlarged in *alk1* mutants. Also note the poor perfusion of brain blood vessels in *eng*$^{-/-}$ fish. Upper right markings indicate the number of fish displaying similar features over the number of fish analyzed. Bar, 100 µm
